# Discovery of Homobivalent Bitopic Ligands of the Cannabinoid CB_2_ Receptor**

**DOI:** 10.1002/chem.202003389

**Published:** 2020-11-09

**Authors:** Paula Morales, Gemma Navarro, Marc Gómez‐Autet, Laura Redondo, Javier Fernández‐Ruiz, Laura Pérez‐Benito, Arnau Cordomí, Leonardo Pardo, Rafael Franco, Nadine Jagerovic

**Affiliations:** ^1^ Medicinal Chemistry Institute Spanish Research Council Madrid Spain; ^2^ Department of Biochemistry and Physiology, CIBERNED Faculty of Pharmacy and Food Sciences Universitat de Barcelona Barcelona Spain; ^3^ Laboratory of Computational Medicine, Biostatistics Unit Faculty of Medicine Universitat Autónoma de Barcelona Barcelona Spain; ^4^ Department of Biochemistry and Molecular Biology, CIBERNED, IRYCIS Faculty of Medicine Universidad Complutense de Madrid Madrid Spain; ^5^ Department of Biochemistry and Molecular Biology, CIBERNED School of Chemistry Universitat de Barcelona Barcelona Spain; ^6^ Present address: Computational Chemistry Janssen Research & Development, Janssen Pharmaceutica N.V. Belgium

**Keywords:** bitopic ligands, CB2 cannabinoid, G protein-coupled receptors, molecular dynamics, site-directed mutagenesis

## Abstract

Single chemical entities with potential to simultaneously interact with two binding sites are emerging strategies in medicinal chemistry. We have designed, synthesized and functionally characterized the first bitopic ligands for the CB_2_ receptor. These compounds selectively target CB_2_ versus CB_1_ receptors. Their binding mode was studied by molecular dynamic simulations and site‐directed mutagenesis.

## Graphical abstract

**Single chemical entities** with potential to simultaneously interact with two binding sites are emerging strategies in GPCR pharmacology. We present herein the design, synthesis and functional characterization of the first bitopic ligands for the CB_2_ receptor. Molecular understanding of the binding mode was guided by molecular dynamic simulations and site‐directed mutagenesis.

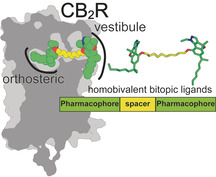

G protein‐coupled receptors (GPCRs) regulate a vast amount of cellular processes,^[1]^ thus, they form one of the most important pharmaceutical drug‐target class (475 drugs in the market that represent ∼34 % of all drugs approved by the US Food and Drug Administration).^[2]^ However, these drugs target only 108 unique GPCRs that are <15 % of the ∼800 genes (or ∼30 % of the ∼360 non‐olfactory GPCRs).^[2]^ One of the reasons is that the orthosteric binding site for a particular endogenous ligand is often highly conserved across a GPCR subfamily, thus making it difficult to achieve high selectivity for specific receptor subtypes.^[3]^ Novel approaches to overcome this problem include the discovery of bitopic ligands that bind the orthosteric site as well as a less conserved site within the same receptor unit.[^4^, ^5^, ^6^] This type of complementary cavity is often located at the entrance of the orthosteric binding site, as identified in ligand binding pathway simulations, which have been named extracellular vestibule^[7]^ or entrance,^[8]^ or secondary^[9]^ or metastable^[10]^ binding site, or exosite.^[11]^ In this work, we will name this cavity as receptor vestibule or exosite. Bitopic ligands that target the orthosteric site and the receptor vestibule improve selectivity,[^11^, ^12^] off‐rates and signaling bias,[^4^, ^13^, ^14^] maintaining bioavailability and brain penetration properties in mice.^[15]^ Other type of comparable ligands are designed to simultaneously bind two orthosteric sites of a (homo/hetero) GPCR dimer.^[16]^ These type of ligands have been recently reviewed.^[17]^

While most GPCRs recognize polar ligands, GPCRs for lipid mediators are activated by hormone‐like signaling molecules derived from lipid species, which possess long hydrophobic moieties. This subfamily is mostly composed of the sphingosine‐1‐phosphate (S1P), lysophosphatidic acid (LPA) and cannabinoid (CB_1_R and CB_2_R) receptors.^[18]^ In the crystal structures of these receptors^[19]^ the extracellular N‐terminus and extracellular loop 2 folds over the ligand binding pocket blocking the access to the orthosteric binding cavity from the extracellular environment. These structures together with binding pathway simulations suggest that binding of lipid‐like ligands to a lipid GPCR occurs through a narrow channel between transmembrane helices (TMs) 1 and 7 that connects the orthosteric binding site to the lipid bilayer.^[20]^ Notably, the access to the ligand pocket of the MT1 melatonin receptor that binds polar ligands (serotonin‐derived compounds) is also via the lipid bilayer.^[21]^ Thus, the design of bitopic ligands for lipid GPCRs is more challenging than for other GPCRs that fully expose the binding site to the extracellular environment, due to the narrow channel linking the binding site and the lipid bilayer.

In the present communication, we have designed bitopic ligands for the cannabinoid CB_2_R. We have selected CB_2_R, instead of CB_1_R, due to its lack of adverse psychotropic effects along with its wide therapeutic application in pathologies such as cancer, neuroinflammation and pain.^[22]^ Bivalent ligands have already been reported for CB_1_R[^23^, ^24^, ^25^] and CB_2_R.^[26]^ These ligands were reported before the release of crystal structures, thus, their binding characteristics remain unclear.[^27^, ^28^] Here, we have used the recently released structure of CB_2_R in its active^[29]^ G_i_‐bound conformation to identify the binding mode of the designed ligands.

The design of bitopic ligands requires the selection of a moiety able to bind the orthosteric site (pharmacophore). In this regard, we have selected chromenopyrazole derivatives A and B (Figure 1), which have been previously identified as CB_2_R orthosteric agonists.^[30]^ Then, a second pharmacophore unit needs to be developed for the vestibule or exosite. This is challenging because this additional cavity has not been properly characterized yet for most GPCRs. We have taken advantage of the simulated binding process of a lipid inhibitor to the S1P1 receptor.^[20]^ The process consists of the diffusion of the ligand through the bilayer leaflet to contact the vestibule at the top of TM 7 (the rate‐limiting step), subsequently moving from this lipid‐facing vestibule to the orthosteric binding cavity through the channel between TMs 1 and 7. We propose that lipid GPCRs are capable to recognize orthosteric ligands at the vestibule of the receptor. Thus, we have also selected the chromenopyrazole moiety as the second pharmacophore so that the designed bitopic ligands are symmetrical (it contains two copies of the same pharmacophore). In addition, an appropriate length spacer to cover the distance between both pharmacophores is required. Importantly, this approach has been recently supported in the model of the bitopic ligand CTL01‐05‐B‐A05, a symmetrical agomelatine molecule linked by an ethoxyethane spacer, which binds both the orthosteric binding site and the exosite of the MT1 melatonin receptor.^[21]^

**Figure 1:**
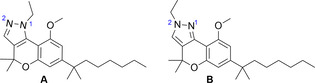
Structures of chromenopyrazoles A and B.^[30]^ These compounds are isomers differing in the position of the *N*‐ethyl at the pyrazole (N1‐ or N2‐ethyl).

Chromenopyrazole A, in its most stable conformation (Figure S1), was docked into the orthosteric and vestibule sites of CB_2_R, and its stability was assessed by molecular dynamic (MD) simulations (Figure S2). Results showed that pharmacophore units remain highly stable at the orthosteric site and moderately stable at the exosite. Visual inspection of the models shows that the −CH_3_ group of the methoxy moieties of both pharmacophores are suitable attachment points to link the spacer moiety. Linker lengths from six to sixteen methylene units were chosen for the bivalent molecules.

Bivalent chromenopyrazoles and their monovalent analogues were synthesized, starting from chromenopyrazoles **4** and **5** (Scheme 1).^[31]^ Preparation of 9‐alkoxychromenopyrazoles (**6**–**17**) was achieved in high yields by deprotonation of the hydroxyl group with sodium hydride, followed by rapid addition of an excess of the appropriate 1‐bromoalkane. Bivalent compounds **18**–**29** were achieved by alkylation of the corresponding chromenopyrazoles with 0.5 equivalents of the desired dibromoalkanes. Different bases were tested, and finally cesium carbonate was selected and used under inert atmosphere. Thus, the desired bivalent compounds (**18**–**29**) were achieved in low to moderate yields.

**Scheme 1:**
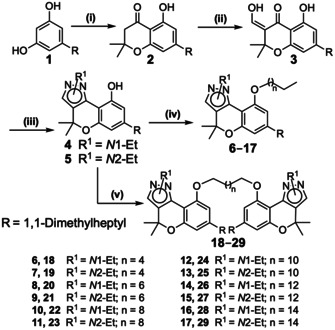
Synthesis of 9‐alkoxychromenopyrazoles **6**–**17** and bivalent chromenopyrazoles **18**–**29**. Reagents and conditions: (i) 3,3‐dimethylacrylic acid, methanesulfonic acid, P_2_O_5_, 8 h, 70 °C, 81 %; (ii) a) NaH, THF, MW, 25 min, 45 °C; b) ethyl formate, MW, 25 min, 45 °C, 76 %; (iii) corresponding hydrazine, EtOH, 1–4 h, 40 °C, 28–74 %; (iv) a) NaH, anhydrous THF, 10 min, b) 1‐bromoalkane, reflux, 2–12 h, 32–75 %; (v) a) Cs_2_CO_3_, anhydrous THF, 10 min, b) 1,(*n*+2)‐dibromoalkane, reflux, 8–72 h, 6–59 %.

In vitro binding affinities of the alleged bitopic ligands **18**–**29** (Table 1) and the corresponding monovalent counterparts **6**–**17** (Table S1) were obtained from [^3^H]CP‐55,940 competition‐binding assays using membrane fractions of the human CB_1_R and CB_2_R, respectively, expressed in HEK‐293T cells. None of the monovalent chromenopyrazoles exhibits affinity towards any of the cannabinoid receptors. Addition of the second pharmacophore makes bivalent ligands capable of binding CB_2_R in a selective manner. Optimal spacer length for N1‐ and N2‐ethyl derivatives is from 10 (*n*=8) to 14 (*n*=12) methylene units.

**Table 1:** Binding affinities of bivalent chromenopyrazoles (**18**–**29**) for *h*CB_1_R and *h*CB_2_R.

Compd	R^1^	*N* ^[a]^	CB_1_R K_i_ [μm]^[b]^	CB_2_R K_i_ [μm]^[b]^
**18**	*N*1‐Et	4	>40	28.1±1.6
**19**	*N*2‐Et	4	>40	12.4±2.0
**20**	*N*1‐Et	6	>40	2.2±0.7
**21**	*N*2‐Et	6	nd	nd
**22**	*N*1‐Et	8	>40	0.9±0.2
**23**	*N*2‐Et	8	>40	5.8±1.5
**24**	*N*1‐Et	10	>40	0.4±0.14
**25**	*N*2‐Et	10	>40	0.3±0.1
**26**	*N*1‐Et	12	>40	0.8±0.1
**27**	*N*2‐Et	12	>40	0.3±0.1
**28**	*N*1‐Et	14	>40	>40
**29**	*N*2‐Et	14	>40	2.12±0.21
**A^30^**	*N*1‐Et	–	5.0±0.7	0.16±0.03
**B^30^**	*N*2‐Et	–	2.9±0.5	0.09±0.02
**WIN** ^[c]^	–	–	0.04±0.01	0.003±0.002

[a] *n* refers to Scheme 1. Total number of methylenes in the spacer is *n*+2. [b] Values obtained from competition curves using [3H]CP55,940 as radioligand for hCB1R and hCB2R and are expressed as the mean±SEM of at least three experiments. nd: not determined. [c] WIN55,212,2.

Compounds with CB_2_R affinity constants in the low micromolar range (**22**, **24**–**27**) were selected for functional evaluation by measuring their effect on forskolin‐induced cAMP levels in HEK‐293 cells expressing hCB_2_R (Figure 2 A). Dose‐response experiments demonstrated that these compounds are able to inhibit cAMP accumulation as efficiently as CP55,940 but with a slight drop of potency (**27**: pEC_50_=7.6 vs. CP55,940: pEC_50_=8.2) (Table 2), while their monovalent counterparts are inactive (Figure S3). None of the compounds displayed an effect in non‐transfected HEK‐293T cells, confirming that the results are fully mediated by CB_2_R (Figure S4).

**Figure 2:**
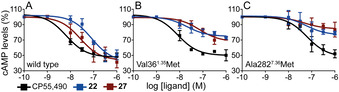
Decrease of forskolin‐induced cAMP (normalized to 100 %), in HEK‐293T cells, upon stimulation of wild type CB_2_R (A) and Val36^1.35^Met (B) and Ala282^7.36^Met (C) mutant receptors with the CP55,940 agonist and ligands **22** and **27**.

**Table 2:** Functional properties of compounds **22**, **24**–**27** and the reference compound CP55,940 at wild type and mutant CB_2_R.

	Wild type		Val36^1.35^Met		Ala282^7.36^Met	
	pEC_50_	*E*_max_^[a]^	pEC_50_	*E*_max_^[a]^	pEC_50_	*E*_max_^[a]^
**22**	7.1±0.2	42±5.4	7.7±0.1	75±1.5	7.2±0.2	77±2.5
**24**	7.2±0.2	45±6.3	nd		nd	
**25**	7.2±0.2	40±5.9	nd		nd	
**26**	6.5±0.2	52±4.1	nd		nd	
**27**	7.6±0.1	44±3.7	7.1±0.2	68±3.5	7.3±0.2	84±1.4
**CP** ^[b]^	8.2±0.1	48±1.7	8.1±0.1	50±2.3	7.2±0.1	48±3.43

[a] *E*_max_ (%), the maximum inhibition of forskolin‐stimulated cAMP levels (normalized to 100 %), values were calculated using nonlinear regression analysis. Data are expressed as the mean±SEM of at least three independent experiments performed in triplicates. [b] CP is CP55,940. nd: not determined.

In order to assess the binding mode of homobivalent chromenopyrazoles **22**, **25** and **27** (10, 12 and 14 methylene units), we docked their most favorable conformation (Figure S1) into CB_2_R in its active state in such a manner that both pharmacophoric units bind into the orthosteric and vestibule sites (Figure 3 A). Unbiased MD simulations show that these chain lengths can simultaneously bind both sites (Figure 3, S5). The methylene spacer expands toward the lipid‐facing vestibule interacting with the hydrophobic side chains of Phe91^2.61^, Ala282^7.36^, Met286^7.40^, and Val36^1.35^. In the vestibule, the chromenopyrazole moiety forms aromatic–aromatic interactions with Phe283^7.37^. In the simulations Phe283^7.37^ adopts the *trans* conformation that opens the channel between TMs 1 and 7 and permits the ligand to reach the membrane, in contrast to the *gauche* ^*+*^ conformation observed in the crystal structure of CB_2_R[^29^, ^32^] that closes the channel. The heptyl chain is accommodated in a hydrophobic cavity of TMs 1 and 7, facing the membrane, which is formed by Leu39^1.38^, Cys40^1.39^, Met286^7.40^, Leu43^1.39^, Ile290^7.44^, Met293^7.47^ and Leu46^1.45^. In the case of the N1‐ethyl derivative the lone pair in the N2 atom forms a hydrogen bond with Gln32^1.31^. A detailed description of these interactions is shown in Figure S6.

**Figure 3:**
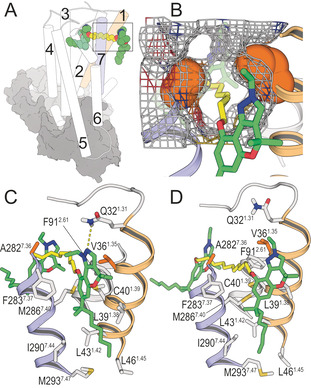
A) General view of the binding mode of bitopic ligand **22** into the orthosteric site and the vestibule of the CB_2_R‐G_i_ complex (depicted as cylinders for CB_2_R and grey surfaces for G_i_). B) Mesh surface of Val36^1.35^ and Ala282^7.36^ (spheres in orange) that formed the channel between TMs 1 and 7. C, D) Detailed views of the binding mode of **22** (C) and **25** (D) into the receptor vestibule obtained during the MD simulations (the longer spacer of **27** permits higher flexibility at the vestibule and some of these interactions are not observed, Figure S6). TMs 1 and 7 are shown in orange and blue, respectively; Val36^1.35^ and Ala282^7.36^, which were mutated to Met, are shown in orange; and the pharmacophore units and spacer of bitopic ligands are shown in green and yellow, respectively.

Figure 3 B shows the mesh surface formed by Val36^1.35^ and Ala282^7.36^. Clearly, these TMs side chains, which are located midway between the orthosteric site and the receptor vestibule, delimit the channel between TMs 1 and 7. Thus, in order to validate the proposed binding mode of bitopic ligands, we mutated the side chains of Val36^1.35^ (Figure 2 B) and Ala282^7.36^ (Figure 2 C) to the much larger Met side chain. As expected, these mutations do not influence the function of the orthosteric agonist CP55,490, but clearly impair signaling of bitopic ligands **22** and **27** (Table 2) by occupying the volume of the channel. Notably, these results contrast with other MD simulations, suggesting the entrance of the ligands from the extracellular environment.[^33^, ^34^]

In summary, we present herein the discovery of CB_2_R homobivalent bitopic ligands. These compounds were designed as symmetrical bivalent compounds using the previously reported chromenopyrazole scaffold as potential pharmacophore for both units linked by a methylene spacer. Binding and functional cAMP studies revealed their ability to selectively activate CB_2_R versus CB_1_R. The longer Ile^1.35^ in CB_1_R than Val^1.35^ in CB_2_R seems responsible for the observed selectivity (Figure S7). MD simulations and site‐directed mutagenesis studies show that these bitopic ligands bind into the orthosteric site and in a vestibule/exosite located at the ligands entry/egress channel that connects the orthosteric site with the lipid bilayer membrane. Whether these bitopic ligands at CB_2_R show beneficial therapeutic application needs further investigation such as structure–activity relationship studies to improve potency.

## Conflict of interest

The authors declare no conflict of interest.

## Supporting information

As a service to our authors and readers, this journal provides supporting information supplied by the authors. Such materials are peer reviewed and may be re‐organized for online delivery, but are not copy‐edited or typeset. Technical support issues arising from supporting information (other than missing files) should be addressed to the authors.

Supplementary
