# Supplementary material for: Discovery of Homobivalent Bitopic Ligands of the Cannabinoid CB2 Receptor
Source: Chemistry. 2020 Nov 9;26(68):15839–42. doi: 10.1002/chem.202003389 (PMC7756656; doi:10.1002/chem.202003389)
Supplement: Supplementary file 1 — Supplementary [file CHEM-26-15839-s001.pdf]

Chemistry–A European Journal

Supporting Information

Discovery of Homobivalent Bitopic Ligands of the Cannabinoid CB_2 Receptor**

Paula Morales+,[a] Gemma Navarro+,[b] Marc Gómez-Autet+,[c] Laura Redondo,[a]
Javier Fernández-Ruiz,[d] Laura Pérez-Benito,[c, f] Arnau Cordomí,[c] Leonardo Pardo,*[c]
Rafael Franco,*[b, e] and Nadine Jagerovic*[a]

TABLE OF CONTENTS

Table S1. Binding affinities of alkoxychromenopyrazoles (**6-17**) for *h*CB_1R and *h*CB_2R.

Figure S1. *Ab initio* conformational analyses of chromenopyrazole derivatives A and B.

Figure S2. MD simulation of chromenopyrazole derivative A docked into the orthosteric site between TMs 2-3, 5-6 and the lipid-facing vestibule in TMs 1 and 7.

Figure S3. Functional assays of selected bivalent compounds in HEK293 cells

Figure S4. Functional assays in non-transfected HEK293 cells.

Figure S5. MD simulations of bitopic ligands **22**, **25**, and **27**.

Figure S6. Heatmap depicting the interactions of bitopic ligands **22**, **25**, and **27** with CB_2R.

Figure S7. Multiple sequence alignment of the amino acids forming the channel between TMs 1 and 7 of GPCRs for lipid mediators.

Experimental Section

1. Chemistry
   - 1.1 General methods and materials
   - 1.2 Synthetic procedures and compounds characterization
2. Pharmacological assays
   - 2.1 Radioligand binding assays
   - 2.2 cAMP determination assays
   - 2.3 CB_2R punctual mutations
3. Molecular modeling
   - 3.1 Docking studies
   - 3.2 Molecular dynamic simulations

References

Table S1. Binding affinities of alkoxychromenopyrazoles (**6**-**17**) for hCB_1R and hCB_2R.

Compd	R^1	n^a	CB_1R K_i [μM]^b	CB_2R K_i [μM]^b
**6**	*N*1-Et	4	>40	>40
**7**	*N*2-Et	4	>40	>40
**8**	*N*1-Et	6	>40	>40
**9**	*N*2-Et	6	>40	>40
**10**	*N*1-Et	8	>40	>40
**11**	*N*2-Et	8	>40	>40
**12**	*N*1-Et	10	>40	>40
**13**	*N*2-Et	10	>40	>40
**14**	*N*1-Et	12	>40	>40
**15**	*N*2-Et	12	>40	>40
**16**	*N*1-Et	14	>40	>40
**17**	*N*2-Et	14	>40	>40

[a]n refers to scheme 2. Total number of methylenes in the spacer is n+2. [b]Values obtained from competition curves using [^{3}H]CP55,940 as radioligand for hCB_1R and hCB_2R and are expressed as the mean ± SEM of at least three experiments.

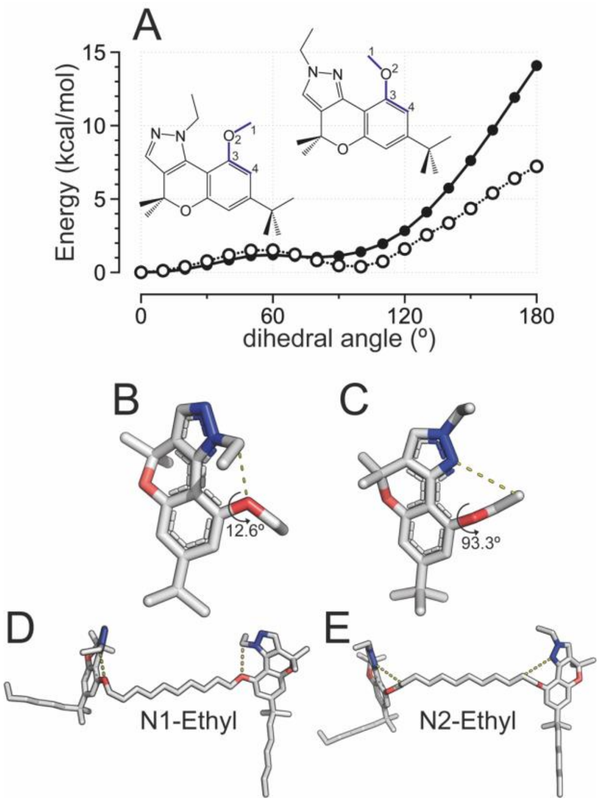

Figure S1. (A) *Ab initio* torsional energy profile (HF/6-31G*//HF/6-31G*) of the C1-O2-C3-C4 dihedral angle (in blue) for chromenopyrazole derivatives A (N1-Ethyl, black circle) and B (N2-Ethyl, white circle) in which the hexyl chains were substituted by methyl. (B, C) In order to properly characterize the orientation of the spacer, the two energy minima (dihedral angles of ~0° and ~90°), obtained in panel A, were used as starting points for *ab initio* full geometry optimization (MP2/6-31G*) of analogous compounds in which $-OCH_3$ was substituted by $-OCH_2CH_3$. The most favorable conformation of the N1-Ethyl analog corresponds to a dihedral angle of 12.6° to favor an intramolecular hydrogen bond (in yellow) between the oxygen atom of the $-OCH_2CH_3$ group and the -CH group of the N1-attached ethyl moiety (B). In contrast, the most favorable conformation of the N2-Ethyl analog corresponds to a dihedral angle of 93.3° to favor an intramolecular hydrogen bond (in yellow) between the -CH- group of $-OCH_2CH_3$ and the N1 atom of the chromenopyrazole moiety (C). (D, E) These most stable conformations, obtained in panels B and C, were used for *ab initio* full geometry optimization (HF/6-31G*) of derivatives **22** (N1-Ethyl, n=8, panel D), **25** (N2-Ethyl, n=10, panel E) and **27** (N2-Ethyl, n=12, not shown), and for deriving RESP atomic charges (HF/6-31G*). This different intramolecular hydrogen bond of the methoxy moiety, to which the methylene spacer is attached, in the N1- and N2-Ethyl derivatives explains the difference in the optimal spacer length (Tables 1 and 2). The calculations were performed with Gaussian 09.

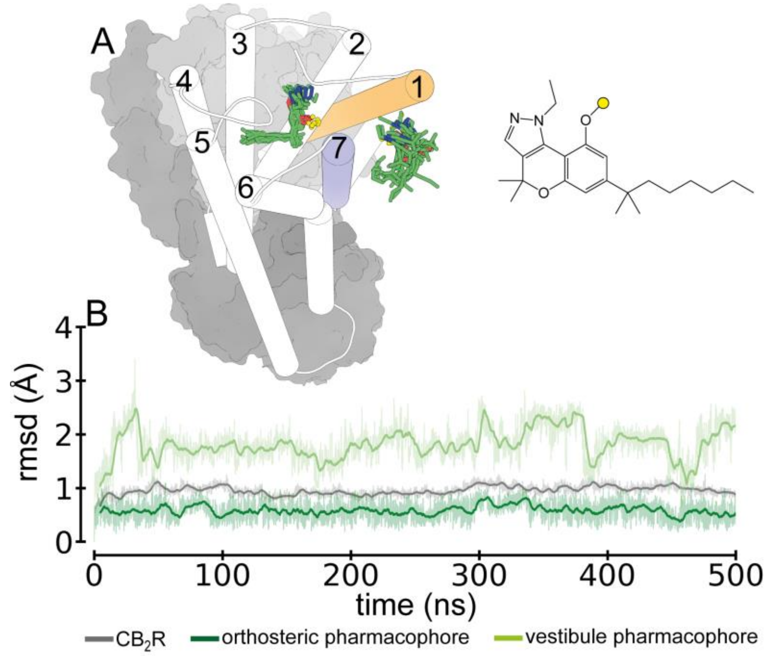

Figure S2. (A) MD simulation of chromenopyrazole derivative A (docked into the orthosteric site between TMs 2-3, 5-6 and the lipid-facing vestibule in TMs 1 and 7) bound to CB_2R (TM helices are depicted as cylinders and loops as ribbons) in complex with G_i (α- and $\beta\gamma$-subunits in dark and light gray surfaces, respectively). The structures of derivative A are extracted from the simulations (10 structures collected every 50 ns), whereas the structure of CB_2R-G_i complex corresponds to the initial structure. (B) The MD simulation was monitored by the root mean-square deviation (rmsd) of the backbone atoms of the TM helices of CB_2R (gray) and heavy atoms of the pharmacophore moieties located at the orthosteric (dark green) and vestibule (light green) binding sites. These results showed that pharmacophore units remain highly stable at the orthosteric site and moderately stable at the vestibule. The proposed attachment points (yellow sphere) are self-oriented in space and can be used for linking the spacer group. Details of the MD simulation are given below in section 3.2.

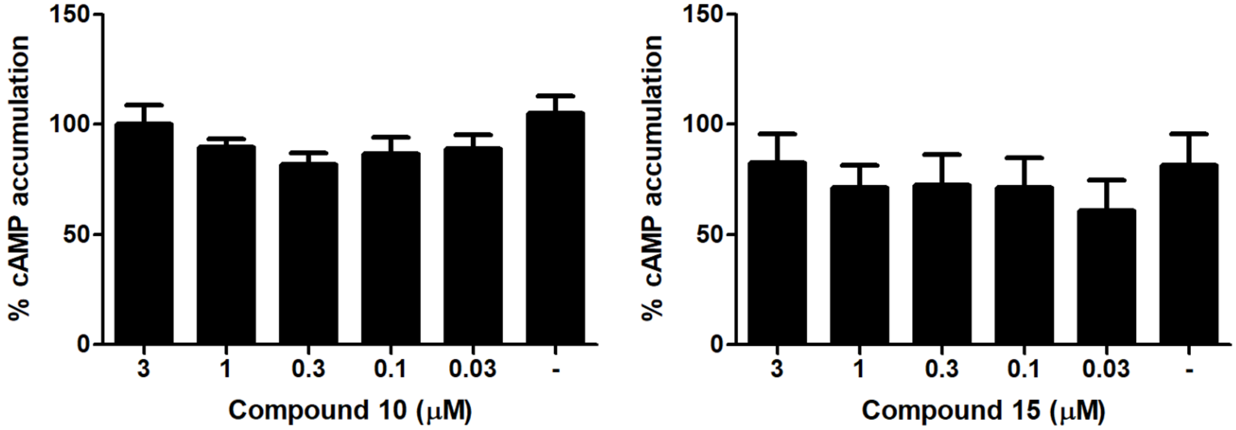

Figure S3. cAMP dose-response assays in CB_2R-HEK293 cells of monovalent chromenopyrazoles **10** and **15**. Compound **10** is the monovalent counterpart of the bitopic ligand **22** and **15** corresponds to bivalent **27**.

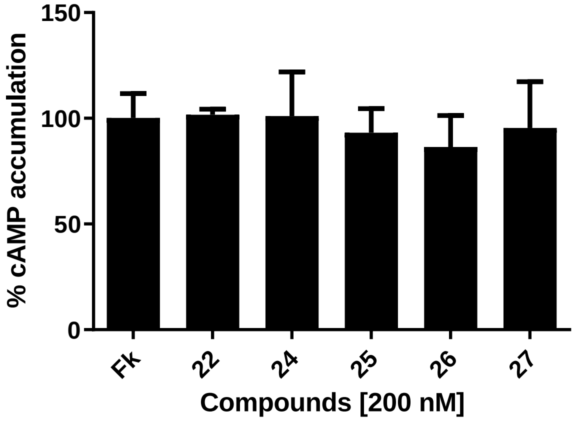

Figure S4. Functional assays in non-transfected HEK293 cells. cAMP screening of bivalent compounds **22**, **24**–**27** in HEK293 cells. Results are expressed as percent of forskolin-stimulated cAMP accumulation at a concentration of 200 nM. All data result from at least three independent experiments, performed in triplicates.

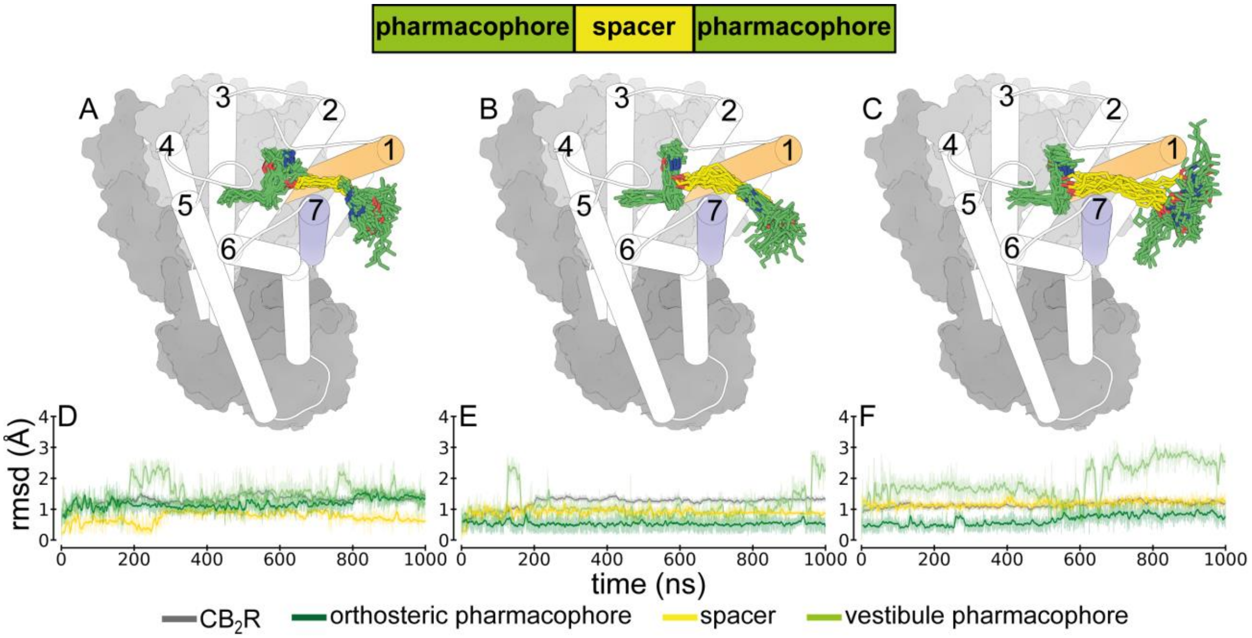

Figure S5. Evolution of homobivalent bitopic ligands **22** (A, D), **25** (B, E) and **27** (C, F) as devised from unbiased 1μs MD simulations (see section 3.2 for computational details and Figure S1 for their *ab initio* full geometry optimization). One replica is displayed for better visualization although an additional replica showed consistent behavior. The structures of **22, 25** and **27** are extracted from the simulations (10 structures collected every 100 ns), whereas the structure of CB_2R (TM helices are depicted as cylinders and loops as ribbons) in complex with G_i (α- and βγ-subunits in dark and light gray surfaces, respectively) corresponds to the initial structure. The stabilities of the ligand-receptor complexes are analyzed via root mean-square deviations (rmsd) of the backbone atoms of the TM helices of CB_2R (gray), heavy atoms of the pharmacophore moieties located at the orthosteric (dark green) and vestibule (light green) binding sites, and the spacer (yellow), in panels D, E and F. As expected, rmsd values of the pharmacophore group at the vestibule are larger than those of the orthosteric site due to a more open, less restrictive, binding cavity. Clearly, rmsd values of the pharmacophore group at the vestibule are larger for compound **27** with 14 methylene units (n=12) as spacer than for compounds **22** with 10 methylene units (n=8) or **25** with 12 methylene units (n=10). The longer spacer of **27** permits higher flexibility at the vestibule.

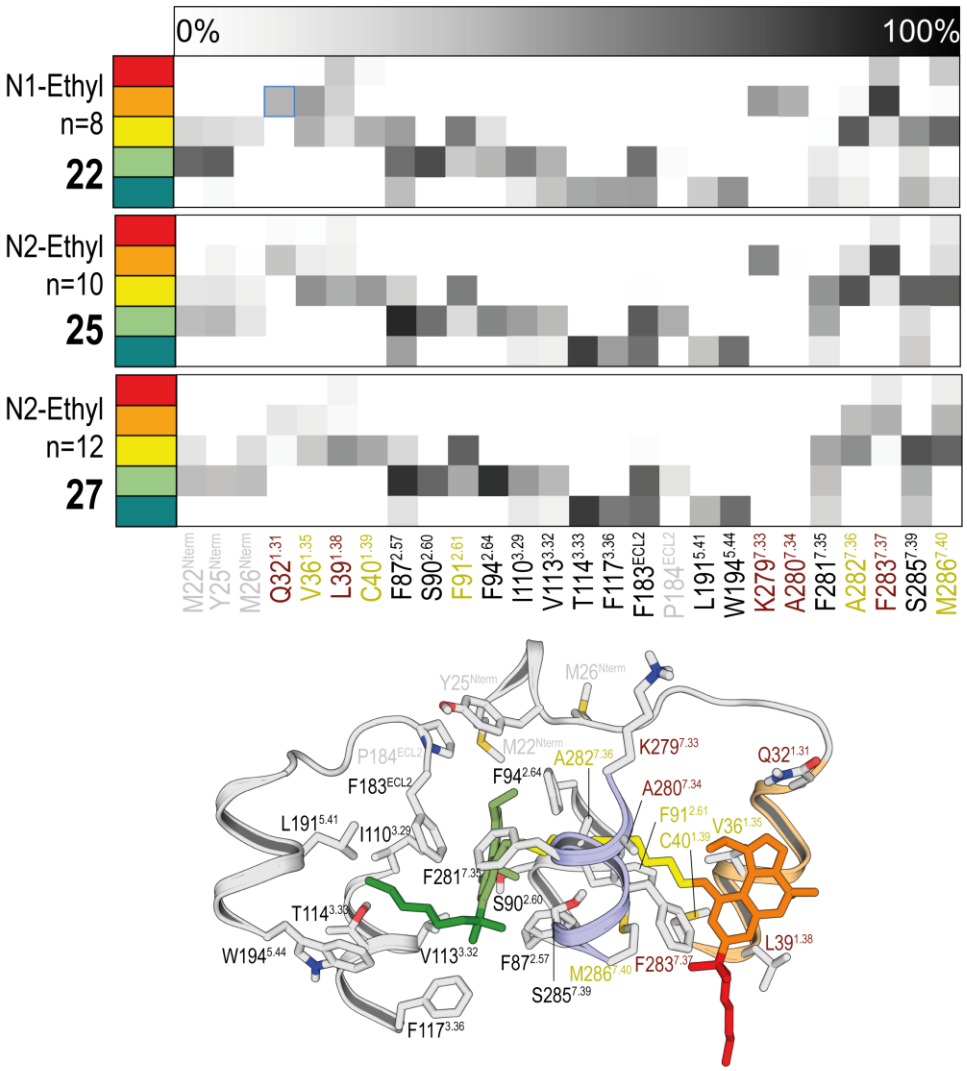

Figure S6. Heatmaps depicting the Van der Waals interactions of bitopic ligands **22** (N1-Ethyl, n=8, 10 methylene units), **25** (N2-Ethyl, n=10, 12 methylene units), and **27** (N2-Ethyl, n=12, 14 methylene units) with amino acids of CB_2R. In order to determine the most important residues of CB_2R that interact with the ligand, the ligand-receptor interactions were quantified during the 6x1µs (two replicas per ligand) of unrestrained MD simulation (see section 3.2 for computational details and Figure S5). The interactions were quantified with GetContacts (https://getcontacts.github.io/interactions.html). Average frequencies have been calculated as the mean proportion between replicas of frames in which the interaction occurs, according to GetContacts interaction criteria. Average frequencies of van der Waals interactions are shown using a grey gradient while consistent hydrogen bond interactions are pinpointed with a blue square. The color code for the moieties of the ligand is: the heptyl and chromenopyrazole moieties of the orthosteric pharmacophore in dark and light green, respectively, the spacer in yellow, and the heptyl and chromenopyrazole moieties of the vestibule pharmacophore in red and orange, respectively. The color code for CB_2R is: the amino acids of the orthosteric binding site are labeled in black, the amino acids that interact with the spacer are labeled in yellow, the amino acids of the vestibule binding site are labeled in red, and the amino acids at the extracellular part defining the cavity in gray. A representative model of bitopic ligand **22** and all the side chains of CB_2R (in white sticks) is depicted. TMs 1 and 7 are shown in light orange and blue, respectively.

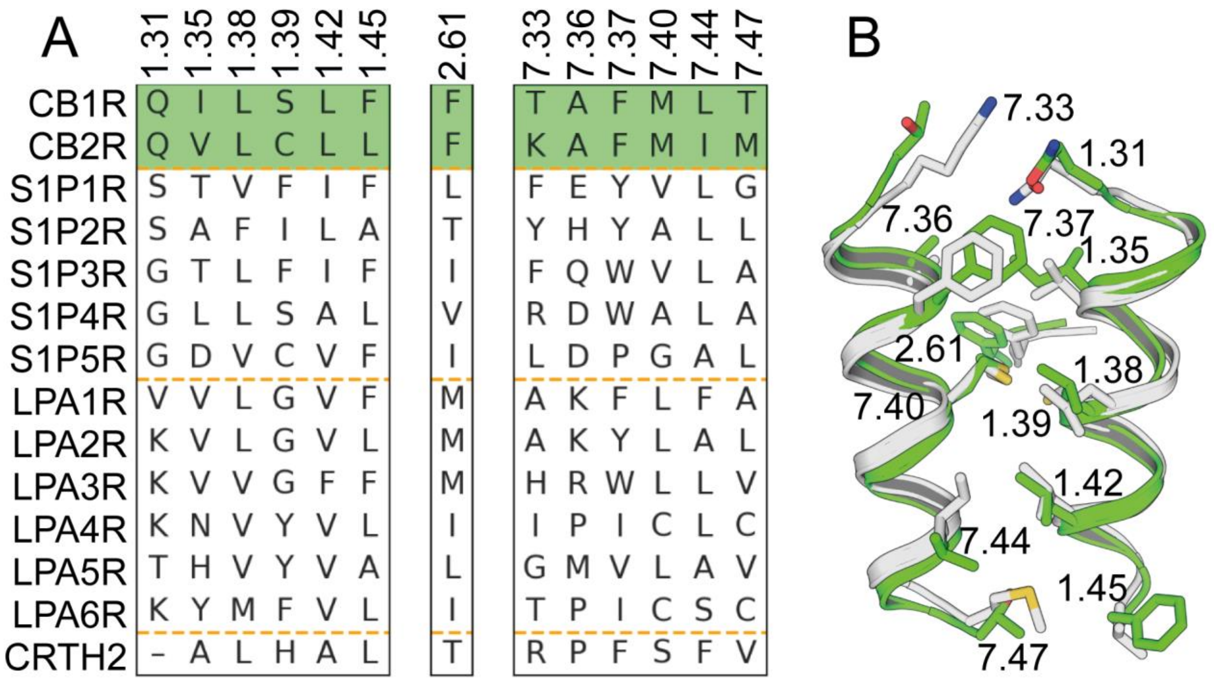

Figure S7. (A) Multiple sequence alignment, among CB_1R and CB_2R, the sphingosine-1-phosphate (S1P1R-S1P5R), lysophosphatidic acid (LPA1R-LPA6R) and prostaglandin D_2 (CRTH2) receptors, of the amino acids forming the channel between TMs 1 and 7. (B) Location of these amino acids in the crystal structures of CB_1R-G_i (PDB id 6N4B, in green) and CB_2R-G_i (PDB id 6PT0, in white) complexes. Addition of the second pharmacophore, which interacts with these amino acids, makes bivalent ligands fully selective toward CB_2R (Table 1). The major change between CB_1R and CB_2R is at position 7.33 (Thr in CB_1R and Lys in CB_2R), however, this amino acid does not frequently interact with all ligands (Figure S6). A minor change is at the key position 1.35 (Figures 2-3, Table 2), the β-branched and very rigid Val side chain in CB_2R, and the likewise β-branched, but longer, Ile in CB_1R. We propose that this additional methyl group of Ile in CB_1R crashes with the bivalent ligand, which explains the observed selectivity.

Experimental Section

1. Chemistry

1.1- *General methods and materials.* Reagents and solvents were purchased from Sigma-Aldrich Co., Fluorochem, Acros Organics, Manchester Organics and Lab-Scan and were used without further purification or drying. Silica gel 60 F254 (0.2 mm) thin layer plates were purchased from Merck GmbH. Products were purified using flash column chromatography (Merck Silica gel 60, 230-400 mesh) or medium pressure chromatography using Biotage Isolera One with pre-packed silica gel columns (Biotage SNAP cartridges). The compounds were characterized by a combination of NMR experiments, and high-resolution mass spectrometry (HRMS). The purity of compounds was determined by LC coupled to HRMS. The experiments were performed in a LC-MS hybrid quadrupole/time of flight (QTOF) analyzer equipped with an Agilent 1200 LC coupled to an Agilent 6500 Accurate Mass (1-2 ppm mass accuracy) using electrospray ionization in the positive mode (ESI+). ^{1}H, ^{13}C, HSQC and HMBC-NMR spectra were recorded on a Varian 500 (500 and 126 MHz) at 25 °C. Samples were prepared as solutions in deuterated solvent and referenced to internal non-deuterated solvent peak. Chemical shifts were expressed in ppm (δ) downfield of tetramethylsilane. Coupling constants are given in hertzs (Hz). The synthesis of compounds **2**–**5** have been previously described by us.[1] A detailed characterization of compounds **6–29** is provided herein. Monovalent and bivalent chromenopyrazoles atoms have been numbered as detailed in compounds **6** and **18** respectively.

1.2 *Synthetic procedures and compounds characterization*

General procedure for the synthesis of alkoxychromenopyrazoles 6–17. The corresponding chromenopyrazole (1eq) was dissolved in anhydrous THF (1-5 mL); the solution was added over sodium hydride (1.5-3 eq) under nitrogen atmosphere. After 10 minutes stirring at room temperature, an excess of the corresponding 1-bromoalkane (5-10 eq) was rapidly added to the solution and the mixture was refluxed (2-12 h). After completion of the reaction (determined by TLC), the mixture was dissolved in

EtOAc, washed with water and extracted three times with EtOAc. The combined organic layers were dried over $MgSO_4$ and the solvent was removed under vacuum. The crude was purified by gradient flash column chromatography with hexane and EtOAc.

7-(1,1-Dimethylheptyl)-1-ethyl-9-hexyloxy1,4-Dihydro-4,4-dimethylchromeno[4,3-*c*]pyrazole (6). Compound **6** was prepared from **4** (30 mg, 0.081 mmol), sodium hydride (5 mg, 0.20 mmol) and 1-bromohexane (80 µL, 0.57 mmol). A yellow oil was obtained (21 mg, 58%). ^{1}H-NMR (500 MHz, $CDCl_3$) δ:

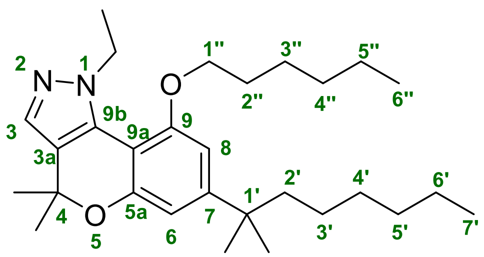

7.32 (s, 1H, 3-H), 6.66 (d, *J* = 1.7 Hz, 1H, 8-H), 6.54 (d, *J* = 1.8 Hz, 1H, 6-H), 4.53 (q, *J* = 7.2 Hz, 2H, NC*H₂*CH₃), 4.08 (t, *J* = 7.0 Hz, 2H, 1''-H), 1.85 (p, *J* = 7.2 Hz, 2H, 2''-H), 1.58 – 1.52 (m, 8H, $OC(CH_3)_2$, *alkyl chain*) , 1.47 – 1.43 (m, 2H, 2'-H), 1.38 (t, *J* = 7.2 Hz, 3H, $NCH_2C\mathit{H_3}$), 1.36 – 1.31 (m, 4H, *alkyl chain*), 1.27 (s, 6H, $C(CH_3)_2$), 1.24 – 1.16 (m, 6H, *alkyl chain*), 1.12 – 1.03 (m, 2H, 3'-H), 0.93 – 0.87 (m, 3H, 6''-H), 0.83 ppm (t, *J* = 7.0 Hz, 2H, 7'-H); ^{13}C-NMR (126 MHz, $CDCl_3$) δ: 153.9 (9-C), 153.4 (7-C), 152.2 (5a-C), 132.2 (9b-C), 132.1 (3-C), 123.3 (3a-C), 109.5 (6-C), 104.6 (8-C), 103.7 (9a-C), 76.4 (4-C), 69.0 (1''-C), 47.9 ($N\underline{C}H_2CH_3$), 44.4 (2'-C), 38.0 (1'-C), 31.7, 31.6, 29.9, 29.2, 25.8, 22.6 and 22.6 (*alkyl chain*), 28.8 ($C(CH_3)_2$), 27.2 ($OC(CH_3)_2$), 24.6 (3'-C), 15.5 ($NCH_2\underline{C}H_3$), 14.1 (7'-C), 14.0 ppm (6''-C); HRMS calcd for $C_{29}H_{47}N_2O_2$ $[M+H]^+$: 455.3632, found: 455.3631.

7-(1,1-Dimethylheptyl)-2-ethyl-9-hexyloxy-2,4-dihydro-4,4-dimethylchromeno[4,3-*c*]pyrazole (7). Compound **7** was prepared from **5** (32 mg, 0.086 mmol), sodium hydride (5.1 mg, 0.22 mmol) and 1-bromohexane (85 µL, 0.60 mmol). A yellow oil was obtained (25 mg, 64%). ^{1}H-NMR (500 MHz, $CDCl_3$) δ: 7.11 (s, 1H, 3-H), 6.55 (d, *J* = 1.6 Hz, 1H, 8-H), 6.52 (d, *J* = 1.7 Hz, 1H, 6-H), 4.19 (q, *J* = 7.3 Hz, 2H, $NC\mathit{H_2}CH_3$), 4.10 (t, *J* = 6.6 Hz, 2H, 1''-H), 1.92 (p, *J* = 6.6 Hz, 2H, 2''-H), 1.66 – 1.60 (m, 6H, *alkyl chain*), 1.58 (s, 6H, $OC(CH_3)_2$), 1.56 – 1.49 (m, 3H, $NCH_2C\mathit{H_3}$), 1.41 – 1.34 (m, 4H, 2'-H, *alkyl chain*), 1.26 (s, 6H, $C(CH_3)_2$), 1.25 – 1.14 (m, 4H, *alkyl chain*), 1.08 – 0.98 (m, 2H, 3'-H), 0.98 – 0.87 (m, 3H, 6''-H), 0.83 ppm (t, *J* = 7.1 Hz, 3H, 7'-H); ^{13}C-NMR (126 MHz, $CDCl_3$) δ: 155.1 (9-C), 153.8 (7-C), 151.1 (5a-C), 140.9 (9b-C),

121.8 (3-C), 121.4 (3a-C), 108.4 (6-C), 105.5 (8-C), 103.9 (9a-C), 75.5 (4-C), 68.9 (1''-C), 47.0 (N<u>C</u>H_2CH_3), 44.6 (2'-C), 38.0 (1'-C), 31.8, 31.7, 30.0, 29.4, 25.8, 22.7 and 22.6 (*alkyl chain*), 28.9 (OC(<u>C</u>$H_3)_2$), 28.8 (C(<u>C</u>$H_3)_2$), 24.6 (3'-C), 15.4 (NCH_2<u>C</u>H_3), 14.1 (7'-C), 14.0 ppm (6''-C); HRMS calcd for $C_{29}H_{47}N_2O_2$ $[M+H]^+$: 455.3632, found: 455.3641.

7-(1,1-Dimethylheptyl)-1-ethyl-1,4-dihydro-4,4-dimethyl-9-octyloxychromeno[4,3-*c*]pyrazole (8). Compound **8** was prepared from **4** (26 mg, 0.070 mmol), sodium hydride (4 mg, 0.17 mmol) and 1-bromooctane (85 µL, 0.49 mmol). A white oil was obtained (23 mg, 70%). ^{1}H-NMR (500 MHz, $CDCl_3$) δ: 7.32 (s, 1H, 3-H), 6.66 (d, *J* = 1.7 Hz, 1H, 8-H), 6.54 (d, *J* = 1.8 Hz, 1H, 6-H), 4.54 (q, *J* = 7.2 Hz, 2H, N*CH₂*CH₃), 4.08 (t, *J* = 7.0 Hz, 2H, 1''-H), 1.85 (p, *J* = 7.0 Hz, 2H, 2''-H), 1.57 – 1.53 (m, 10H, $OC(CH_3)_2$, *alkyl chain*), 1.47 – 1.43 (m, 2H, 2'-H), 1.38 (t, *J* = 7.2 Hz, 3H, $NCH_2C\mathit{H_3}$), 1.34 – 1.25 (m, 14H, $C(CH_3)_2$, *alkyl chain*), 1.23 – 1.16 (m, 6H, *alkyl chain*), 1.12 – 1.02 (m, 2H, 3'-H), 0.88 (t, *J* = 6.8 Hz, 3H, 8''-H), 0.83 ppm (t, *J* = 7.0 Hz, 3H, 7'-H); ^{13}C-NMR (126 MHz, $CDCl_3$) δ: 153.9 (9-C), 153.4 (7-C), 152.2 (5a-C), 132.2 (9b-C), 132.1 (3-C), 123.3 (3a-C), 109.5 (6-C), 104.6 (8-C), 103.7 (9a-C), 76.4 (4-C), 69.0 (1''-C), 47.9 ($N\mathit{C}H_2CH_3$), 44.4 (2'-C), 38.0 (1'-C), 31.8, 31.7, 29.9, 29.4, 29.3, 29.2, 26.1, 22.63 and 22.61 (*alkyl chain*), 28.8 ($C(\mathit{C}H_3)_2$), 27.2 ($OC(\mathit{C}H_3)_2$), 24.6 (3'-C), 15.5 ($NCH_2\mathit{C}H_3$), 14.1 (7'-C), 14.0 ppm (8''-C); HRMS calcd for $C_{31}H_{51}N_2O_2$ $[M+H]^+$: 483.3945, found: 483.3969.

7-(1,1-Dimethylheptyl)-2-ethyl-2,4-dihydro-4,4-dimethyl-9-octyloxychromeno[4,3-*c*]pyrazole (9). Compound **9** was prepared from **5** (21 mg, 0.057 mmol), sodium hydride (3 mg, 0.14 mmol) and 1-bromooctane (68 µL, 0.39 mmol). A yellow oil was obtained (17 mg, 63%). ^{1}H-NMR (500 MHz, $CDCl_3$) δ: 7.12 (s, 1H, 3-H), 1.56 (d, *J* = 1.7 Hz, 1H, 8-H), 6.52 (d, *J* = 1.7 Hz, 1H, 6-H), 4.20 (q, *J* = 7.4 Hz, 2H, $N\mathit{CH_2}CH_3$), 4.10 (t, *J* = 6.7 Hz, 2H, 1''-H), 1.92 (p, *J* = 6.7 Hz, 2H, 2''-H), 1.58 (s, 6H, $OC(CH_3)_2$), 1.64 – 1.59 (m, 6H, *alkyl chain*), 1.53 (t, *J* = 7.3 Hz, 3H, $NCH_2C\mathit{H_3}$), 1.43 – 1.27 (m, 6H, 2'-H, *alkyl chain*), 1.26 (s, 6H, $C(CH_3)_2$), 1.24 – 1.13 (m, 6H, *alkyl chain*), 1.08 – 1.00 (m, 2H, 3'-H), 0.88 (t, *J* = 6.8 Hz, 3H, 8''-H), 0.83

ppm (t, J = 7.0 Hz, 3H, 7'-H); ^{13}C-NMR (126 MHz, $CDCl_3$) δ: 155.1 (9-C), 153.8 (7-C), 151.1 (5a-C), 140.9 (9b-C), 121.7 (3-C), 121.4 (3a-C), 108.4 (6-C), 103.9 (9a-C), 75.5 (4-C), 69.0 (1''-C), 47.0 (NCH$_2$CH$_3$), 44.6 (2'-C), 38.0 (1'-C), 31.9, 31.8, 30.0, 29.6, 29.5, 29.4, 26.07, 22.70 and 22.62 (*alkyl chain*), 28.9 (OC(CH$_3$)$_2$), 28.8 (C(CH$_3$)$_2$), 24.6 (3'-C), 15.4 (NCH$_2$$CH_3$), 14.1 (7'-C), 14.0 ppm (8''-C); HRMS calcd for $C_{31}H_{51}N_2O_2$ [M+H]$^+$: 483.3946, found: 483.3969.

9-Decyloxy-7-(1,1-dimethylheptyl)-1-ethyl-1,4-dihydro-4,4-dimethylchromeno[4,3-*c*]pyrazole (10). Compound **10** was prepared from **4** (14 mg, 0.038 mmol), sodium hydride (2 mg, 0.095 mmol) and 1-bromodecane (55 µL, 0.27 mmol). A white oil was obtained (12 mg, 62%). ^{1}H-NMR (500 MHz, $CDCl_3$) δ: 7.32 (s, 1H, 3-H), 6.66 (d, J = 1.7 Hz, 1H, 8-H), 6.54 (d, J = 1.7 Hz, 1H, 6-H), 4.53 (q, J = 7.2 Hz, 2H, NCH_2CH$_3$), 4.08 (t, J = 7.0 Hz, 2H, 1''-H), 1.85 (p, J = 7.0 Hz, 2H, 2''-H), 1.55 (s, 6H, OC(CH$_3$)$_2$), 1.48–1.40 (m, 2H, 2'-H), 1.38 (t, J = 7.2 Hz, 3H, NCH$_2$CH_3), 1.33–1.21 (m, 20H, *alkyl chain*), 1.22–1.15 (m, 6H, C(CH$_3$)$_2$), 1.11–1.04 (m, 2H, 3'-H), 0.88 (t, J = 6.8 Hz, 3H, 10''-H), 0.84 ppm (t, J = 7.0 Hz, 3H, 7'-H); ^{13}C-NMR (126 MHz, $CDCl_3$) δ: 153.9 (9-C), 153.4 (7-C), 152.1 (5a-C), 132.2 (9b-C), 132.0 (3-C), 123.2 (3a-C), 109.5 (6-C), 104.6 (8-C), 103.7 (9a-C), 76.4 (OC(CH$_3$)$_2$), 69.0 (1''-C), 47.9 (NCH$_2$CH$_3$), 44.4 (2'-C), 38.0 (C(CH$_3$)$_2$), 31.9, 31.7, 29.9, 29.54, 29.52, 29.4, 29.3, 29.2, 26.1, 22.7 and 22.6 (*alkyl chain*), 28.7 (C(CH$_3$)$_2$), 27.2 (OC(CH$_3$)$_2$), 24.6 (3'-C), 15.5 (NCH$_2$$CH_3$), 14.1 (7'-C), 14.0 ppm (10''-C); HRMS calcd for $C_{33}H_{55}N_2O_2$ [M+H]$^+$: 511.4258, found: 511.4260.

9-Decyloxy-7-(1,1-dimethylheptyl)-2-ethyl-2,4-dihydro-4,4-dimethylchromeno[4,3-*c*]pyrazole (11). Compound **11** was prepared from **5** (18 mg, 0.048 mmol), sodium hydride (2.9 mg, 0.12 mmol) and 1-bromodecane (70 µL, 0.34 mmol). A pale-yellow oil was obtained (18 mg, 75%). ^{1}H-NMR (500 MHz, $CDCl_3$) δ: 7.11 (s, 1H, 3-H), 6.56 (d, J = 1.7 Hz, 1H, 8-H), 6.52 (d, J = 1.7 Hz, 1H, 6-H), 4.19 (q, J = 7.3 Hz, 2H, NCH_2CH$_3$), 4.09 (t, J = 6.6 Hz, 2H, 1''-H), 1.91 (p, J = 6.6 Hz, 2H, 2''-H), 1.58 (s, 6H, OC(CH$_3$)$_2$), 1.52 (t, J = 7.3 Hz, 3H, NCH$_2$CH_3), 1.43–1.33 (m, 2H, 2'-H), 1.32 – 1.22 (br s, 20H, C(CH$_3$)$_2$, *alkyl chain*), 1.19–1.13 (m, 6H, *alkyl chain*), 1.09–0.97 (m, 2H, 3'-H), 0.87 (t, J = 6.8, 3H, 10''-H), 0.83 ppm (t, J = 7.0 Hz, 3H, 7'-H);

^{13}C-NMR (126 MHz, $CDCl_3$) δ: 155.0 (9-C), 153.7 (7-C), 151.0 (5a-C), 140.9 (9b-C), 121.7 (3-C), 121.3 (3a-C), 108.4 (6-C), 105.6 (8-C), 103.9 (9a-C), 75.5 (O*C*(CH_3)$_2$), 68.9 (1''-C), 47.0 (N*C*H_2CH_3), 44.6 (2'-C), 37.9 (*C*(CH_3)$_2$), 31.9, 31.7, 29.9, 29.7, 29.6, 29.5, 29.3, 26.1, 22.7 and 22.6 (*alkyl chain*), 28.9 (OC(*C*H_3)$_2$), 28.8 (C(*C*H_3)$_2$), 24.6 (3'-C), 15.4 (NCH_2*C*H_3), 14.1 (7'-C), 14.0 ppm (10''-C); HRMS calcd for $C_{33}H_{55}N_2O_2$ $[M+H]^+$: 511.4258, found: 511.4277.

7-(1,1-Dimethylheptyl)-9-dodecyloxy-1-ethyl-1,4-dihydro-4,4-dimethylchromeno[4,3-*c*]pyrazole (12). **12** was prepared from **4** (30 mg, 0.08 mmol), sodium hydride (6 mg, 0.24 mmol) and 1-bromododecane (0.19 mL, 0.81 mmol). A yellow oil was obtained (17 mg, 39%). ^{1}H-NMR (500 MHz, $CDCl_3$) δ: 7.32 (s, 1H, 3-H), 6.66 (d, *J* = 1.7 Hz, 1H, 8-H), 6.54 (d, *J* = 1.7 Hz, 1H, 6-H), 4.53 (q, *J* = 7.2 Hz, 2H, NC*H*$_2$$CH_3$), 4.08 (t, *J* = 7.0 Hz, 2H, 1''-H), 1.85 (p, *J* = 7.2 Hz, 2H, 2''-H), 1.59–1.51 (m, 10H, OC(CH_3)$_2$, *alkyl chain*), 1.46–1.40 (m, 2H, 2'-H), 1.38 (t, *J* = 7.2 Hz, 3H, NCH_2C*H*$_3$), 1.28–1.26 (m, 10H, *alkyl chain*), 1.26–1.25 (m, 12H, C(CH_3)$_2$, *alkyl chain*), 1.21–1.18 (m, 4H, *alkyl chain*), 1.10–1.03 (m, 2H, 3'-H), 0.88 (t, *J* = 6.9 Hz, 3H, 12''-H), 0.84 ppm (t, *J* = 6.8 Hz, 3H, 7'-H); ^{13}C-NMR (126 MHz, $CDCl_3$) δ: 153.9 (9-C), 153.4 (7-C), 152.2 (5a-C), 132.2 (9b-C), 132.1 (3-C), 123.3 (3a-C), 109.5 (6-C), 104.6 (8-C), 103.7 (9a-C), 76.4 (O*C*(CH_3)$_2$), 69.0 (1''-C), 47.9 (N*C*H_2CH_3), 44.4 (2'-C), 38.0 (*C*(CH_3)$_2$), 31.9, 31.7, 29.9, 29.7, 29.6, 29.58, 29.56, 29.46, 29.3, 29.2, 26.1, 22.7 and 22.6 (*alkyl chain*), 28.8 (C(*C*H_3)$_2$), 27.2 (OC(*C*H_3)$_2$), 24.6 (3'-C), 15.5 (NCH_2*C*H_3), 14.1 (7'-C), 14.0 ppm (12''-C); HRMS calcd for $C_{35}H_{59}N_2O_2$ $[M+H]^+$: 539.4571, found: 539.4556.

7-(1,1-Dimethylheptyl)-9-dodecyloxy-2-ethyl-2,4-dihydro-4,4-dimethylchromeno[4,3-*c*]pyrazole (13). Compound **13** was prepared from **5** (42 mg, 0.11 mmol), sodium hydride (7 mg, 0.28 mmol) and 1-bromododecane (0.19 mL, 0.79 mmol). A yellow oil was obtained (45 mg, 74%). ^{1}H-NMR (500 MHz, $CDCl_3$) δ: 7.11 (s, 1H, 3-H), 6.56 (d, *J* = 1.6 Hz, 1H, 8-H), 6.52 (d, *J* = 1.6 Hz, 1H, 6-H), 4.19 (q, *J* = 7.3 Hz, 2H, NC*H*$_2$$CH_3$), 4.09 (t, *J* = 6.6 Hz, 2H, 1''-H), 1.91 (p, *J* = 6.6 Hz, 2H, 2''-H), 1.62 (p, *J* = 7.1 Hz, 2H, *alkyl chain*), 1.58 (s, 6H, OC(CH_3)$_2$), 1.53 (t, *J* = 7.3 Hz, 3H, NCH_2C*H*$_3$), 1.43–1.35 (m, 2H, 2'-H), 1.27–1.25 (br s, 22H, C(CH_3)$_2$, *alkyl chain*), 1.19 – 1.14 (m, 6H, *alkyl chain*), 1.07 – 1.00 (m, 2H, 3'-H), 0.88 (t, *J* = 7.1, 3H, 12''-

H), 0.83 ppm (t, J = 7.0 Hz, 3H, 7'-H); ^{13}C-NMR (126 MHz, $CDCl_3$) δ: 155.1 (9-C), 153.7 (7-C), 151.0 (5a-C), 140.9 (9b-C), 121.8 (3-C), 121.4 (3a-C), 108.4 (6-C), 105.6 (8-C), 104.0 (9a-C), 75.5 (O*C*(CH_3)$_2$), 67.0 (1''-C), 47.0 (N*C*H_2CH_3), 44.6 (2'-C), 38.0 (*C*(CH_3)$_2$), 31.9, 31.8, 30.0, 29.72, 29.71, 29.68, 29.65, 29.64, 29.5, 29.4, 26.1, 22.7 and 22.6 (*alkyl chain*), 28.9 (OC(*C*H_3)$_2$), 28.8 (C(*C*H_3)$_2$), 24.6 (3'-C), 15.4 (NCH_2*C*H_3), 14.1 (7'-C), 14.0 ppm (12''-C); HRMS calcd for $C_{35}H_{59}N_2O_2$ $[M+H]^+$: 539.4571, found: 539.4578.

7-(1,1-Dimethylheptyl)-1-ethyl-1,4-dihydro-4,4-dimethyl-9-tetradecyloxychromeno[4,3-*c*]pyrazole (14). Compound **14** was prepared from **4** (16.5 mg, 0.045 mmol), sodium hydride (3 mg, 0.11 mmol) and 1-bromotetradecane (94 µL, 0.32 mmol). A yellow oil was obtained (19 mg, 75%). ^{1}H-NMR (500 MHz, $CDCl_3$) δ: 7.32 (s, 1H, 3-H), 6.66 (d, J = 1.7 Hz, 1H, 8-H), 6.54 (d, J = 1.7 Hz, 1H, 6-H), 4.53 (q, J = 7.2 Hz, 2H, NC*H*$_2CH_3$), 4.08 (t, J = 7.0 Hz, 2H, 1''-H), 1.85 (p, J = 7.0 Hz, 2H, 2''-H), 1.55 (s, 6H, OC(CH_3)$_2$), 1.47–1.40 (m, 2H, 2'-H), 1.38 (t, J = 7.2 Hz, 3H, NCH_2C*H*$_3$), 1.28 (s, 6H, C(CH_3)$_2$), 1.27–1.24 (m, 22H, *alkyl chain*), 1.23–1.16 (m, 6H, *alkyl chain*), 1.11–1.03 (m, 2H, 3'-H), 0.88 (t, J = 6.8 Hz, 3H, 14''-H), 0.84 ppm (t, J = 7.2 Hz, 3H, 7'-H); ^{13}C-NMR (126 MHz, $CDCl_3$) δ: 153.9 (9-C), 153.4 (7-C), 152.1 (5a-C), 132.2 (9b-C), 132.0 (3-C), 123.2 (3a-C), 109.5 (6-C), 104.6 (8-C), 103.7 (9a-C), 76.3 (O*C*(CH_3)$_2$), 69.0 (1''-C), 47.9 (N*C*H_2CH_3), 44.4 (2'-C), 38.0 (*C*(CH_3)$_2$), 31.9, 31.7, 29.9, 29.7, 29.63, 29.58, 29.55, 29.4, 29.3, 29.2, 26.1, 22.7 and 22.6 (*alkyl chain*), 28.7(C(*C*H_3)$_2$), 27.2 (OC(*C*H_3)$_2$), 24.6 (3'-C), 15.5 (NCH_2*C*H_3), 14.1 (7'-C), 14.0 ppm (14''-C); HRMS calcd for $C_{37}H_{63}N_2O_2$ $[M+H]^+$: 567.4884, found: 567.4898.

7-(1,1-Dimethylheptyl)-2-ethyl-2,4-dihydro-4,4-dimethyl-9-tetradecyloxychromeno[4,3-*c*]py-razole (15). Compound **15** was prepared from **5** (23 mg, 0.06 mmol), sodium hydride (3.7 mg, 0.15 mmol) and 1-bromotetradecane (0.13 mL, 0.43 mmol). A yellow oil was obtained (23 mg, 66%). ^{1}H-NMR (500 MHz, $CDCl_3$) δ: 7.11 (s, 1H, 3-H), 6.56 (d, J = 1.7 Hz, 1H, 8-H), 6.52 (d, J = 1.7 Hz, 1H, 6-H), 4.19 (q, J = 7.3 Hz, 2H, NC*H*$_2CH_3$), 4.09 (t, J = 6.7 Hz, 2H, 1''-H), 1.92 (p, J = 6.7 Hz, 2H, 2''-H), 1.58 (s, 6H, OC(CH_3)$_2$), 1.53 (t, J = 7.3 Hz, 3H, NCH_2C*H*$_3$), 1.45–1.32 (m, 8H, 2'-H, *alkyl chain*), 1.26–1.24 (br s, 22H, C(CH_3)$_2$, *alkyl chain*), 1.20–1.13 (m, 6H, *alkyl chain*), 1.08–1.00 (m, 2H, 3'-H), 0.88 (t, J = 6.7, 3H, 14''-H), 0.83 ppm (t, J = 7.0

Hz, 3H, 7'-H); ^{13}C-NMR (126 MHz, $CDCl_3$) δ: 155.0 (9-C), 153.7 (7-C), 151.0 (5a), 140.9 (9b-C), 121.7 (3-C), 121.3 (3a-C), 108.4 (6-C), 105.5 (8-C), 103.9 (9a-C), 75.4 (O*C*(CH_3)$_2$), 68.9 (1''-C), 47.0 (N*C*H_2CH_3), 44.6 (2'-C), 37.9 (*C*(CH_3)$_2$), 31.9, 31.7, 29.9, 29.71, 29.70, 29.65, 29.63, 29.5, 29.4, 22.7 and 22.6 (*alkyl chain*), 28.9 (OC(*C*H_3)$_2$), 28.8 (C(*C*H_3)$_2$), 24.5 (3'-C), 15.4 (NCH_2*C*H_3), 14.1 (7'-C), 14.0 ppm (14''-C); HRMS calcd for $C_{37}H_{63}N_2O_2$ $[M+H]^+$: 567.4884, found: 567.4899.

7-(1,1-Dimethylheptyl)-1-ethyl-9-hexadecyloxy-1,4-dihydro-4,4-dimethylchromeno[4,3-*c*]pyrazole (16). Compound **16** was prepared from **4** (50 mg, 0.14 mmol), sodium hydride (10 mg, 0.41 mol) and 1-bromohexadecane (0.21 mL, 1.4 mmol). An orange oil was obtained (46 mg, 57%). ^{1}H-NMR (500 MHz, $CDCl_3$) δ: 7.32 (s, 1H, 3-H), 6.66 (d, *J* = 1.7 Hz, 1H, 8-H), 6.54 (d, *J* = 1.7 Hz, 1H, 6-H), 4.53 (q, *J* = 7.2 Hz, 2H, NC*H*$_2$CH$_3$), 4.08 (t, *J* = 7.0 Hz, 2H, 1''-H), 1.85 (p, *J* = 7.1 Hz, 2H, 2''-H), 1.55 (s, 6H, OC(CH_3)$_2$), 1.46–1.41 (m, 2H, 2'-H), 1.38 (t, *J* = 7.2 Hz, 3H, NCH_2C*H*$_3$), 1.27 (s, 6H, C(CH_3)$_2$), 1.26–1.25 (br s, 24H, *alkyl chain*), 1.22–1.18 (m, 8H, *alkyl chain*), 1.11–1.03 (m, 2H, 3'-H), 0.87 (t, *J* = 6.8 Hz, 3H, 16''-H), 0.83 ppm (t, *J* = 6.6 Hz, 3H, 7'-H); ^{13}C-NMR (126 MHz, $CDCl_3$) δ: 153.9 (9-C), 153.4 (7-C), 152.1 (5a-C), 132.2 (9b-C), 132.0 (3-C), 123.3 (3a-C), 109.5 (6-C), 104.6 (8-C), 103.7 (9a-C), 76.4 (O*C*(CH_3)$_2$), 69.0 (1''-C), 47.9 (N*C*H_2CH_3), 44.4 (2'-C), 38.0 (*C*(CH_3)$_2$), 31.9, 31.7, 29.9, 29.69, 29.65, 29.59, 29.56, 29.5, 29.4, 29.2, 26.1, 22.7 and 22.6 (*alkyl chain*), 28.8 (C(*C*H_3)$_2$), 27.2 (OC(*C*H_3)$_2$), 24.6 (3'-C), 15.5 (NCH_2*C*H_3), 14.1 (7'-C), 14.0 ppm (16''-C); HRMS calcd for $C_{39}H_{67}N_2O_2$ $[M+H]^+$: 595.5197, found: 595.5213.

7-(1,1-Dimethylheptyl)-2-ethyl-9-hexadecyloxy-2,4-dihydro-4,4-dimethylchromeno[4,3-*c*]py-razole (17). Compound **17** was prepared from **5** (45 mg, 0.12 mmol), sodium hydride (9 mg, 0.36 mmol) and 1-bromohexadecane (0.26 mL, 0.85 mmol). A orange oil was obtained (31 mg, 43%). ^{1}H-NMR (500 MHz, $CDCl_3$) δ: 7.11 (s, 1H, 3-H), 6.55 (d, *J* = 1.6 Hz, 1H, 8-H), 6.52 (d, *J* = 1.6 Hz, 1H, 6-H), 4.19 (q, *J* = 7.3 Hz, 2H, NC*H*$_2$CH$_3$), 4.09 (t, *J* = 6.8 Hz, 2H, 1''-H), 1.91 (p, *J* = 6.8 Hz, 2H, 2''-H), 1.65–1.59 (m, 2H, *alkyl chain*), 1.58 (s, 6H, OC(CH_3)$_2$), 1.52 (t, *J* = 7.3 Hz, 3H, NCH_2C*H*$_3$), 1.42–1.34 (m, 2H, 2'-H), 1.27–1.25 (br s, 30H, C(CH_3)$_2$, *alkyl chain*), 1.22–1.10 (m, 6H, *alkyl chain*), 1.04–1.00 (m, 2H, 3'-H), 0.88 (t, *J* = 7.0 Hz, 3H, 16''-H), 0.83

ppm (t, J = 7.0 Hz, 3H, 7'-H); ^{13}C-NMR (126 MHz, $CDCl_3$) δ: 155.1 (9-C), 153.7 (7-C), 151.0 (5a-C), 140.9 (9b-C), 121.8 (3-C), 121.4 (3a-C), 108.4 (6-C), 105.6 (8-C), 104.0 (9a-C), 75.5 (O*C*(CH_3)$_2$), 69.0 (1''-C), 47.0 (N*C*H_2CH_3), 44.6 (2'-C), 38.0 (*C*(CH_3)$_2$), 31.9, 31.8, 30.0, 29.73, 29.71, 29.69, 29.66, 29.65, 29.5, 29.4, 26.1, 22.7 and 22.6 (*alkyl chain*), 28.9 (OC(*C*H_3)$_2$), 28.8 (C(*C*H_3)$_2$), 24.6 (3'-C), 15.4 (NCH_2*C*H_3), 14.1 (7'-C), 14.0 ppm (16''-C); HRMS calcd for $C_{39}H_{67}N_2O_2$ $[M+H]^+$: 595.5197, found: 595.5203.

General procedure the synthesis of bivalent chromenopyrazoles 18-29. A solution of the corresponding chromenopyrazole (1 eq) in anhydrous THF (1-5 mL) was added to a Kimax® vial containing Cs2CO3 (10 eq) under nitrogen atmosphere. After 10 minutes stirring, the cor-respondent dibromoalkane (0.5 eq) was added. The mix-ture was heated at reflux temperature for 8-72 hours. Then, the mixture was dissolved in EtOAc, washed with water and extracted three times with EtOAc. The organic layers were combined and dried over MgSO4, filtered and the solvent was evaporated under reduced pressure. The crude was purified by gradient flash column chromatog-raphy with hexane and EtOAc.

1,6-*Bis*[(7-(1,1-dimethylheptyl)-1-ethyl-1,4-dihydro-4,4-dimethylchromeno[4,3-*c*]pyrazol-9-yl)oxy]hexane (18). Compound **18** was prepared from (26 mg, 0.07 mmol), Cs_2CO_3 (0.23 g, 0.70 mmol) and 1,6-dibromohexane (5 µL, 0.035 mmol). A yellow oil was obtained (8 mg, 14%). ^{1}H-NMR (500 MHz, $CDCl_3$) δ: 7.32 (s, 2H, 3-H), 6.67–6.63 (bs, 2H, 8-H), 6.54–6.50 (bs, 2H, 6-H), 4.53 (q, J = 7.1 Hz, 4H, NC*H*$_2CH_3$), 4.09 (t, J = 6.7 Hz, 4H, 1''-H), 1.95–1.82 (m, 4H, 2''-H), 1.61 – 1.45 (bs, 18H, OC(CH_3)$_2$, 2'-H, *alkyl chain*), 1.37 (t, J = 7.1 Hz, 6H, NCH_2C*H*$_3$), 1.27 (s, 12H, C(CH_3)$_2$), 1.23 – 1.15 (m, 14H, *alkyl chain*), 1.12 – 0.99 (m, 4H, 3'-H), 0.83 ppm (t, J = 6.6 Hz, 6H, 7'-H); ^{13}C-NMR (126 MHz, $CDCl_3$) δ: 153.9 (9-C), 153.3 (7-C), 152.2 (5a-C), 132.3 (9b-C), 132.0 (3-C), 123.3 (3a-C), 109.6 (6-C), 104.6 (8-C), 103.6 (9a-C), 76.4 (4-C), 68.8 (1''-C), 47.8 (N*C*H_2CH_3), 44.4 (2'-C), 38.0 (1'-C), 31.7, 29.9, 29.2, 26.1 and 22.6 (*alkyl*

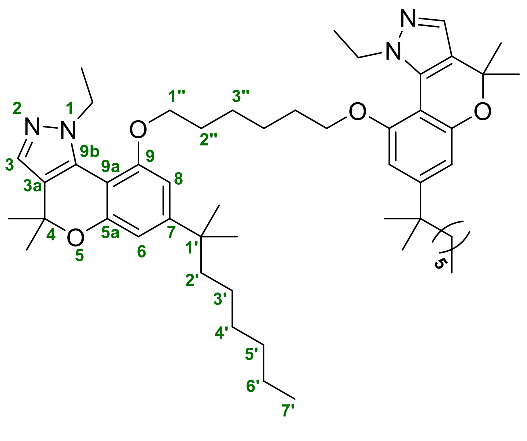

chain), 28.7 (C(*C*H_3)$_2$), 27.2 (OC(*C*H_3)$_2$), 24.6 (3'-C), 15.5 (NCH_2*C*H_3), 14.1 ppm (7'-C); HRMS calcd for $C_{52}H_{79}N_4O_4$ [M+H]$^+$: 823.6096, found: 823.6125.

1,6-*Bis*[(7-(1,1-dimethylheptyl)-2-ethyl-2,4-dihydro-4,4-dimethylchromeno[4,3-*c*]pyrazol-9-yl)oxy]hexane (19). Compound **19** was prepared from **5** (29 mg, 0.078 mmol), Cs_2CO_3 (0.25 g, 0.78 mmol) and 1,6-dibromohexane (6 µL, 0.039 mmol). A yellow oil was obtained (4 mg, 6%). ^{1}H-NMR (500 MHz, $CDCl_3$) δ: 7.11 (s, 2H, 3-H), 6.56 (d, *J* = 1.6 Hz, 2H, 8-H), 6.53 (d, *J* = 1.6 Hz, 2H, 6-H), 4.17 (q, *J* = 7.3 Hz, 4H, NC*H$_2$*CH_3), 4.12 (t, *J* = 6.6 Hz, 4H, 1''-H), 2.00 – 1.94 (m, 4H, 2''-H), 1.58 – 1.54 (bs, 12H, OC(CH_3)$_2$), 1.51 (t, *J* = 7.3 Hz, 6H, NCH_2C*H$_3$*), 1.26 (s, 12H, C(CH_3)$_2$), 1.22 – 1.12 (m, 16H, *alkyl chain*), 1.09 – 0.99 (m, 4H, 3'-H), 0.83 ppm (t, *J* = 7.1 Hz, 6H, 7'-H); ^{13}C-NMR (126 MHz, $CDCl_3$) δ: 155.1 (9-C), 153.8 (7-C), 151.1 (5a-C), 140.9 (9b-C), 121.8 (3-C), 121.4 (3a-C), 108.5 (6-C), 105.6 (8-C), 104.0 (9a-C), 75.5 (4-C), 70.0 (1''-C), 47.0 (N*C*H_2CH_3), 44.6 (2'-C), 38.0 (1'-C), 31.8, 30.0, 29.6, 26.1 and 22.6 (*alkyl chain*), 29.0 (OC(*C*H_3)$_2$), 28.8 (C(*C*H_3)$_2$), 24.6 (3'-C), 15.4 (NCH_2*C*H_3), 14.1 ppm (7'-C); HRMS calcd for $C_{52}H_{79}N_4O_4$ [M+H]$^+$: 823.6096, found: 823.6123.

1,8-*Bis*[(7-(1,1-dimethylheptyl)-1-ethyl-1,4-dihydro-4,4-dimethylchromeno[4,3-*c*]pyrazol-9-yl)oxy]octane (20). Compound **20** was prepared from **4** (21 mg, 0.056 mmol), Cs_2CO_3 (0.18 g, 0.56 mmol) and 1,8-dibromooctane (5 µL, 0.03 mmol). A yellow oil was obtained (5 mg, 11%). ^{1}H-NMR (500 MHz, $CDCl_3$) δ: 7.32 (s, 2H, 3-H), 6.66 (d, *J* = 1.6 Hz, 2H, 8-H), 6.54 (d, *J* = 1.6 Hz, 2H, 6-H), 4.53 (q, *J* = 7.2 Hz, 4H, NC*H$_2$*CH_3), 4.08 (t, *J* = 6.9 Hz, 4H, 1''-H), 1.86 (p, *J* = 7.0 Hz, 4H, 2''-H), 1.55 (s, 16H, OC(CH_3)$_2$), 1.50 – 1.42 (m, 6H, 2'-H, *alkyl chain*), 1.37 (t, *J* = 7.2 Hz, 6H, NCH_2C*H$_3$*), 1.27 (s, 12H, C(CH_3)$_2$), 1.23 – 1.16 (m, 14H, *alkyl chain*), 1.12 – 1.03 (m, 4H, 3'-H), 0.83 ppm (t, *J* = 6.9 Hz, 6H, 7'-H); ^{13}C-NMR (126 MHz, $CDCl_3$) δ: 153.9 (9-C), 153.4 (7-C), 152.2 (5a-C), 132.2 (9b-C), 132.1 (3-C), 123.3 (3a-C), 109.6 (6-C), 104.6 (8-C), 103.6 (9a-C), 76.4 (4-C), 68.9 (1''-C), 47.9 (N*C*H_2CH_3), 44.4 (2'-C), 38.0 (1'-C), 31.7, 29.9, 29.4, 29.2, 26.1

and 22.6 (*alkyl chain*), 28.7 ($C(CH_3)_2$), 27.2 ($OC(CH_3)_2$), 24.6 (3'-C), 15.5 (NCH_2CH_3), 14.1 ppm (7'-C); HRMS calcd for $C_{54}H_{83}N_4O_4$ $[M+H]^+$: 851.6409, found: 851.6393.

1,8-*Bis*[(7-(1,1-dimethylheptyl)-2-ethyl-2,4-dihydro-4,4-dimethylchromeno[4,3-*c*]pyrazol-9-yl)oxy]octane (21). Compound **21** was prepared from **5** (16 mg, 0.043 mmol), Cs_2CO_3 (0.14 g, 0.43 mmol) and 1,8-dibromooctane (4 µL, 0.02 mmol). A yellow oil was obtained (3 mg, 8%). ^{1}H-NMR (500 MHz, $CDCl_3$) δ: 7.11 (s, 2H, 3-H), 6.56 (d, *J* = 1.6 Hz, 2H, 8-H), 6.52 (d, *J* = 1.6 Hz, 2H, 6-H), 4.18 (q, *J* = 7.3 Hz, 4H, NCH_2CH_3), 4.09 (t, *J* = 6.6 Hz, 4H, 1''-H, 1.93 (p, *J* = 6.7 Hz, 4H, 2''-H), 1.69 – 1.61 (m, 4H, *alkyl chain*), 1.58 (s, 12H, $OC(CH_3)_2$), 1.56 – 1.53 (m, 8H, *alkyl chain*), 1.51 (t, *J* = 7.3 Hz, 6H, NCH_2CH_3), 1.47 – 1.45 (m, 8H, 2'-H, *alkyl chain*), 1.26 (s, 12H, $C(CH_3)_2$), 1.24 – 1.13 (m, 17H), 1.07 – 1.00 (m, 4H, 3'-H), 0.83 ppm (t, *J* = 7.1 Hz, 6H, 7'-H); ^{13}C-NMR (126 MHz, $CDCl_3$) δ: 155.1 (9-C), 153.8 (7-C), 151.1 (5a-C), 140.9 (9b-C), 121.7 (3-C), 121.4 (3a-C), 108.5 (6-C), 105.5 (8-C), 103.9 (9a-C), 75.5 (4-C), 68.9 (1''-C), 47.0 (NCH_2CH_3), 44.6 (2'-C), 38.0 (1'-C), 31.8, 30.0, 29.7, 29.5, 26.1 and 22.6 (*alkyl chain*), 28.9 ($OC(CH_3)_2$), 28.8 ($C(CH_3)_2$), 24.6 (3'-C), 15.4 (NCH_2CH_3), 14.1 ppm (7'-C); HRMS calcd for $C_{54}H_{83}N_4O_4$ $[M+H]^+$: 851.6409, found: 851.6399.

1,10-*Bis*[(7-(1,1-dimethylheptyl)-1-ethyl-1,4-dihydro-4,4-dimethylchromeno[4,3-*c*]pyrazol-9-yl)oxy]decane (22). Compound **22** was prepared from **4** (23 mg, 0.062 mmol), Cs_2CO_3 (0.20 g, 0.62 mmol) and 1,10-dibromodecane (9.0 mg, 0.03 mmol). A yellow oil was obtained (12 mg, 44%). ^{1}H-NMR (500 MHz, $CDCl_3$) δ: 7.32 (s, 2H, 3-H), 6.66 (d, *J* = 1.7 Hz, 2H, 8-H), 6.54 (d, *J* = 1.7 Hz, 2H, 6-H), 4.53 (q, *J* = 7.2 Hz, 4H, NCH_2CH_3), 4.08 (t, *J* = 7.1 Hz, 4H, 1''-H), 1.85 (p, *J* = 7.1 Hz, 4H, 2''-H), 1.54–1.57 (m, 24H, $OC(CH_3)_2$, *alkyl chain*), 1.48–1.40 (m, 4H, 2'-H), 1.37 (t, *J* = 7.2 Hz, 6H, NCH_2CH_3), 1.33–1.29 (m, 8H, *alkyl chain*), 1.27–1.25 (br s, 12H, $C(CH_3)_2$), 1.23–1.15 (m, 4H, *alkyl chain*), 1.13–1.02 (m, 4H, 3'-H), 0.82 ppm (t, *J* = 7.0 Hz, 6H, 7'-H); ^{13}C-NMR (126 MHz, $CDCl_3$) δ: 153.9 (9-C), 153.4 (7-C), 152.1 (5a-C), 132.2 (9b-C), 132.0 (3-C), 123.3 (3a-C), 109.5 (6-C), 104.6 (8-C), 103.6 (9a-C), 76.4 ($OC(CH_3)_2$), 69.0 (1''-C), 47.9 (NCH_2CH_3), 44.4 (2'-C), 38.0 ($C(CH_3)_2$), 31.7, 29.9, 29.50, 29.47, 29.2, 26.1 and 22.6 (*alkyl chain*), 28.7

($C(CH_3)_2$), 27.2 ($OC(CH_3)_2$), 24.6 (3'-C), 15.5 (NCH_2CH_3), 14.1 ppm (7'-C); HRMS calcd for $C_{56}H_{87}N_4O_4$ $[M+H]^+$: 879.6722, found: 879.6704.

1,10-*Bis*[(7-(1,1-dimethylheptyl)-2-ethyl-2,4-dihydro-4,4-dimethylchromeno[4,3-*c*]pyrazol-9-yl)oxy]decane (23). Compound **23** was prepared from **5** (29 mg, 0.079 mmol), Cs_2CO_3 (0.26 g, 0.79 mmol) and 1,10-dibromodecane (11.6 mg, 0.04 mmol). A yellow oil was obtained (10 mg, 29%). ^{1}H-NMR (500 MHz, $CDCl_3$) δ: 7.26 (s, 2H, 3-H), 6.56 (d, J = 1.7 Hz, 2H, 8-H), 6.52 (d, J = 1.7 Hz, 2H, 6-H), 4.18 (q, J = 7.3 Hz, 4H, NCH_2CH_3), 4.09 (t, J = 6.6 Hz, 4H, 1''-H), 1.91 (p, J = 6.6 Hz, 4H, 2''-H), 1.57– 1.55 (m, 26H, $OC(CH_3)_2$, *alkyl chain*), 1.51 (t, J = 7.3 Hz, 6H, NCH_2CH_3), 1.42–1.33 (m, 8H, 2'-H, *alkyl chain*), 1.26 (s, 12H, $C(CH_3)_2$), 1.21–1.13 (m, 6H, *alkyl chain*), 1.09–0.98 (m, 4H, 3'-H), 0.83 ppm (t, J = 7.0 Hz, 6H, 7'-H); ^{13}C-NMR (126 MHz, $CDCl_3$) δ: 155.1 (9-C), 153.7 (7-C), 151.0 (5a-C), 140.9 (9b-C), 121.7 (3-C), 121.4 (3a-C), 108.5 (6-C), 105.6 (8-C), 103.9 (9a-C), 75.5 ($OC(CH_3)_2$), 68.9 (1''-C), 47.0 (NCH_2CH_3), 44.6 (2'-C), 38.0 ($C(CH_3)_2$), 31.7, 30.0, 29.73, 29.69, 29.5, 26.1 and 22.6 (*alkyl chain*), 28.9 ($OC(CH_3)_2$), 28.8 ($C(CH_3)_2$), 24.5 (3'-C), 15.4 (NCH_2CH_3), 14.1 ppm (7'-C); HRMS calcd for $C_{56}H_{87}N_4O_4$ $[M+H]^+$: 879.6722, found: 879.6736.

1,12-*Bis*[(7-(1,1-dimethylheptyl)-1-ethyl-1,4-dihydro-4,4-dimethylchromeno[4,3-*c*]pyrazol-9-yl)oxy]dodecane (24). Compound **24** was prepared from **4** (25 mg, 0.07 mmol), Cs_2CO_3 (0.22 g, 0.68 mmol) and 1,12-dibromododecane (10.7 mg, 0.03 mmol). A white oil was obtained (10 mg, 32%). ^{1}H-NMR (500 MHz, $CDCl_3$) δ: 7.32 (s, 2H, 3-H), 6.66 (d, J = 1.7, 2H, 8-H), 6.54 (d, J = 1.7 Hz, 2H, 6-H), 4.53 (q, J = 7.2 Hz, 4H, NCH_2CH_3), 4.08 (t, J = 7.0 Hz, 4H, 1''-H), 1.85 (p, J = 6.9 Hz, 4H, 2''-H), 1.60–1.52 (m, 20H, $OC(CH_3)_2$, *alkyl chain*), 1.47–1.40 (m, 4H, 2'-H), 1.38 (t, J = 7.2 Hz, 6H, NCH_2CH_3), 1.29–1.26 (m, 20H, $C(CH_3)_2$, *alkyl chain*), 1.24–1.17 (m, 12H, *alkyl chain*), 1.11–1.04 (m, 4H, 3'-H), 0.83 ppm (t, J = 6.9 Hz, 6H, 7'-H); ^{13}C-NMR (126 MHz, $CDCl_3$) δ: 153.9 (9-C), 153.4 (7-C), 152.2 (5a-C), 132.2 (9b-C), 132.0 (3-C), 123.3 (3a-C), 109.5 (6-C), 104.6 (8-C), 103.6 (9a-C), 76.4 ($OC(CH_3)_2$), 69.0 (1''-C), 47.9 (NCH_2CH_3), 44.4 (2'-C), 38.0 ($C(CH_3)_2$), 31.7, 30.9, 29.9, 29.6, 29.5, 29.2, 26.1 and 22.6 (*alkyl chain*), 28.8 ($C(CH_3)_2$), 27.2

($OC(CH_3)_2$), 24.6 (3'-C), 15.5 (NCH_2CH_3), 14.1 ppm (7'-C); HRMS calcd for $C_{58}H_{91}N_4O_4$ $[M+H]^+$: 907.7035, found: 907.7042.

1,12-*Bis*[(7-(1,1-dimethylheptyl)-2-ethyl-2,4-dihydro-4,4-dimethylchromeno[4,3-*c*]pyrazol-9-yl)oxy]dodecane (25). Compound **25** was prepared from **5** (52 mg, 0.14 mmol), Cs_2CO_3 (0.46 g, 1.40 mmol) and 1,12-dibromododecane (22.10 mg, 0.07 mmol). A yellow oil was isolated (11 mg, 18%). ^{1}H-NMR (500 MHz, $CDCl_3$) δ: 7.11 (s, 2H, 3-H), 6.56 (d, *J* = 1.6 Hz, 2H, 8-H), 6.52 (d, *J* = 1.6 Hz, 2H, 6-H), 4.18 (q, *J* = 7.3 Hz, 4H, NCH_2CH_3), 4.09 (t, *J* = 6.6 Hz, 4H, 1''-H), 1.96–1.89 (m, 4H, 2''-H), 1.65–1.59 (m, 4H, *alkyl chain*), 1.57 (s, 12H, $OC(CH_3)_2$), 1.52 (t, *J* = 7.3 Hz, 6H, NCH_2CH_3), 1.41–1.28 (m, 20H, 2'-H, *alkyl chain*), 1.26 (s, 12H, $C(CH_3)_2$), 1.20–1.13 (m, 8H, *alkyl chain*), 1.06–1.01 (m, 4H, 3'-H), 0.83 ppm (t, *J* = 7.0 Hz, 6H, 7'-H); ^{13}C-NMR (126 MHz, $CDCl_3$) δ:155.1 (9-C), 153.7 (7-C), 151.0 (5a-C), 140.9 (9b-C), 121.8 (3-C), 121.4 (3a-C), 108.5 (6-C), 105.6 (8-C), 103.9 (9a-C), 75.5 ($OC(CH_3)_2$), 69.0 (1''-C), 47.0 (NCH_2CH_3), 44.6 (2'-C), 38.0 ($C(CH_3)_2$), 31.8, 30.0, 29.8, 29.7, 29.5, 26.1, 24.6 and 22.6 (*alkyl chain*), 28.9 ($OC(CH_3)_2$), 28.8 ($C(CH_3)_2$), 15.4 (NCH_2CH_3), 14.1 ppm (7'-C); HRMS calcd for $C_{58}H_{91}N_4O_4$ $[M+H]^+$: 907.7035, found: 907.7031.

1,14-*Bis*[(7-(1,1-dimethylheptyl)-1-ethyl-1,4-dihydro-4,4-dimethylchromeno[4,3-*c*]pyrazol-9-yl)oxy]tetradecane (26). Compound **26** was prepared from **4** (18 mg, 0.048 mmol), Cs_2CO_3 (0.15 g, 0.48 mmol) and 1,14-dibromotetradecane (8.6 mg, 0.024 mmol). A pale-yellow oil was obtained (9 mg, 39%). ^{1}H-NMR (500 MHz, $CDCl_3$) δ: 7.32 (s, 2H, 3-H), 6.66 (d, *J* = 1.7 Hz, 2H, 8-H), 6.54 (d, *J* = 1.7 Hz, 2H, 6-H), 4.53 (q, *J* = 7.2 Hz, 4H, NCH_2CH_3), 4.08 (t, *J* = 7.0 Hz, 4H, 1''-H), 1.85 (p, *J* = 7.0 Hz, 4H, 2''-H), 1.59–1.53 (m, 22H, $OC(CH_3)_2$, *alkyl chain*), 1.48–1.42 (m, 4H, 2'-H), 1.37 (t, *J* = 7.2 Hz, 6H, NCH_2CH_3), 1.27 (s, 24H, $C(CH_3)_2$, *alkyl chain*), 1.22–1.17 (m, 10H, *alkyl chain*), 1.12–1.06 (m, 4H, 3'-H), 0.83 ppm (t, *J* = 6.9 Hz, 6H, 7'-H); ^{13}C-NMR (126 MHz, $CDCl_3$) δ: 153.9 (9-C), 153.4 (7-C), 152.1 (5a-C), 132.2 (9b-C), 132.0 (3-C), 123.3 (3a-C), 109.5 (6-C), 104.6 (8-C), 103.7 (9a-C), 76.4 ($OC(CH_3)_2$), 69.0 (1''-C), 47.9 (NCH_2CH_3), 44.4 (2'-C), 38.0 ($C(CH_3)_2$), 31.7, 29.9, 29.7, 29.6, 29.57, 29.50, 29.2, 26.1 and 22.6 (*alkyl chain*), 28.8 ($C(CH_3)_2$), 27.2

(OC(CH_3)$_2$), 24.6 (3′-C), 15.5 (NCH$_2$$CH_3$), 14.1 ppm (7′-C); HRMS calcd for $C_{60}H_{95}N_4O_4$ $[M+H]^+$: 935.7347, found: 935.7360.

1,14-*Bis*[(7-(1,1-dimethylheptyl)-2-ethyl-2,4-dihydro-4,4-dimethylchromeno[4,3-*c*]pyrazol-9-yl)oxy]tetradecane (27). Compound **27** was prepared from **5** (20 mg, 0.054 mmol), Cs_2CO_3 (0.17 g, 0.54 mmol) and 1,14-dibromotetradecane (9.6 mg, 0.027 mmol). A yellow oil was obtained (15 mg, 59%). ^{1}H-NMR (500 MHz, $CDCl_3$) δ: 7.10 (s, 2H, 3-H), 6.56 (d, *J* = 1.6 Hz, 2H, 8-H), 6.51 (d, *J* = 1.6 Hz, 2H, 6-H), 4.18 (q, *J* = 7.3 Hz, 4H, NCH_2CH$_3$), 4.09 (t, *J* = 6.6 Hz, 4H, 1′′-H), 1.90 (q, *J* = 7.2, 6.8 Hz, 4H, 2′′-H), 1.60–1.55 (m, 26H, OC(CH_3)$_2$, *alkyl chain*), 1.52 (t, *J* = 7.3 Hz, 6H, NCH$_2$CH_3), 1.33–1.22 (br s, 12H, C(CH_3)$_2$), 1.22–1.11 (m, 22H, *alkyl chain*), 1.06–1.00 (m, 4H, 3′-H), 0.83 ppm (t, *J* = 6.7 Hz, 6H, 7′-H); ^{13}C-NMR (126 MHz, $CDCl_3$) δ: 155.1 (9-C), 153.8 (7-C), 151.0 (5a-C), 140.9 (9b-C), 121.8 (3-C), 121.4 (3a-C), 108.5 (6-C), 105.6 (8-C), 104.0 (9a-C), 75.5 (O*C*(CH_3)$_2$), 69.0 (1′′-C), 47.0 (N*C*H$_2$CH$_3$), 44.6 (2′-C), 38.0 (*C*(CH_3)$_2$), 31.8, 30.0, 29.74, 29.72, 29.69, 29.5, 26.1 and 22.6 (*alkyl chain*), 29.0 (OC(CH_3)$_2$), 28.9 (C(CH_3)$_2$), 24.6 (3′-C), 15.4 (NCH$_2$$CH_3$), 14.1 ppm (7′-C); HRMS calcd for $C_{60}H_{95}N_4O_4$ $[M+H]^+$: 935.7347, found: 935.7359.

1,16-*Bis*[(7-(1,1-dimethylheptyl)-1-ethyl-1,4-dihydro-4,4-dimethylchromeno[4,3-*c*]pyrazol-9-yl)oxy]hexadecane (28). Compound **28** was prepared from **4** (35 mg, 0.095 mmol), Cs_2CO_3 (0.31 g, 0.95 mmol) and 1,16-dibromohexadecane (17.5 mg, 0.048 mmol). An orange oil was isolated (8 mg, 17%). ^{1}H-NMR (500 MHz, $CDCl_3$) δ: 7.33 (s, 2H, 3-H), 6.66 (d, *J* = 1.8 Hz, 2H, 8-H), 6.54 (d, *J* = 1.8 Hz, 2H, 6-H), 4.54 (q, *J* = 7.2 Hz, 4H, NCH_2CH$_3$), 4.08 (t, *J* = 7.0 Hz, 4H, 1′′-H), 1.85 (p, *J* = 7.2 Hz, 4H, 2′′-H), 1.53–1.58 (m, 20H, OC(CH_3)$_2$, *alkyl chain*), 1.46–1.42 (m, 4H, 2′-H), 1.38 (t, *J* = 7.2 Hz, 6H, NCH$_2$CH_3), 1.30–1.23 (m, 28H, C(CH_3)$_2$, *alkyl chain*), 1.22–1.17 (m, 12H, *alkyl chain*), 1.11–1.03 (m, 4H, 3′-H), 0.84 ppm (t, *J* = 6.8 Hz, 6H, 7′-H); ^{13}C-NMR (126 MHz, $CDCl_3$) δ: 153.9 (9-C), 153.4 (7-C), 152.2 (5a-C), 132.1 (9b-C), 132.1 (3-C), 123.3 (3a-C), 109.5 (6-C), 104.6 (8-C), 103.7 (9a-C), 76.3 (O*C*(CH_3)$_2$), 69.0 (1′′-C), 47.9 (N*C*H$_2$CH$_3$), 44.4 (2′-C), 38.1 (*C*(CH_3)$_2$), 31.7, 29.9, 29.70, 29.67, 29.62, 29.58, 29.49, 29.2, 26.1 and 22.6 (*alkyl chain*), 28.8

($C(CH_3)_2$), 27.2 ($OC(CH_3)_2$), 24.6 (3'-C), 15.5 (NCH_2CH_3), 14.1 ppm (7'-C); HRMS calcd for $C_{62}H_{99}N_4O_4$ $[M+H]^+$: 963.7661, found: 963.7635.

1,16-*Bis*[(7-(1,1-dimethylheptyl)-2-ethyl-2,4-dihydro-4,4-dimethylchromeno[4,3-*c*]pyrazol-9-yl)oxy]hexadecane (29). Compound **29** was prepared from **5** (19 mg, 0.051 mmol), Cs_2CO_3 (0.17 g, 0.51 mmol) and 1,16-dibromohexadecane (9.4 mg, 0.025 mmol). A yellow oil was isolated (7 mg, 29%). ^1H-NMR (500 MHz, $CDCl_3$) δ: 7.11 (s, 2H, 3-H), 6.56 (d, *J* = 1.6 Hz, 2H, 8-H), 6.52 (d, *J* = 1.6 Hz, 2H, 6-H), 4.19 (q, *J* = 7.3 Hz, 4H, NCH_2CH_3), 4.09 (t, *J* = 6.6 Hz, 4H, 1''-H), 1.91 (p, *J* = 7.3 Hz, 4H, 2''-H), 1.64–1.59 (m, 8H, *alkyl chain*), 1.57 (s, 12H, $OC(CH_3)_2$), 1.52 (t, *J* = 7.3 Hz, 6H, NCH_2CH_3), 1.42–1.34 (m, 4H, 2''-H), 1.28–1.25 (m, 28H, $C(CH_3)_2$, *alkyl chain*), 1.23–1.13 (m, 12H, *alkyl chain*), 1.08–1.00 (m, 4H, 3''-H), 0.83 ppm (t, *J* = 7.0 Hz, 6H, 7'-H); ^{13}C-NMR (126 MHz, $CDCl_3$) δ: 155.1 (9-C), 153.8 (7-C), 151.0 (5aC), 140.9 (9b-C), 121.7 (3-C), 121.4 (3a-C), 108.4 (6-C), 105.5 (8-C), 104.0 (9a-C), 75.5 ($OC(CH_3)_2$), 69.0 (1''-C), 47.0 (NCH_2CH_3), 44.6 (2'-C), 38.0 ($C(CH_3)_2$), 31.8, 30.0, 29.8, 29.74, 29.71, 29.66, 29.5, 26.1 and 22.6 (*alkyl chain*), 28.9 ($OC(CH_3)_2$), 28.8 ($C(CH_3)_2$), 24.6 (3'-C), 15.5 (NCH_2CH_3), 14.1 ppm (7'-C); HRMS calcd for $C_{62}H_{99}N_4O_4$ $[M+H]^+$: 963.7661, found: 963.7691.

2. Pharmacological assays

2.1- *Radioligand binding assays*. Commercial membranes purified from cells transfected with human CB_1R or CB_2R (RBHCB1M400UA and RBXCB2M400UA) were supplied by PerkinElmer Life and Analytical Sciences (Boston, MA, USA). The protein concentration was 8 µg/well for CB_1R and 4 µg/well for the CB_2R. The binding buffer was 50 mM TrisCl, 5 mM MgCl2, 2.5 mM EDTA, and 0.5 mg/mL BSA (pH 7.4) for CB_1R, and 50 mM TrisCl, 5 mM MgCl2, 2.5 mM EGTA, and 1 mg/mL BSA (pH 7.5) for CB_2R. The radioligand [3H]-CP55940 (PerkinElmer, Boston, MA, USA) was used at a concentration of membrane KD × 0.8 nM, and the final incubation volume was 200 µL for CB_1R and 600 µL for CB_2R receptors. 96-well plates and the tubes necessary for the experiment were previously siliconized with Sigmacote (Sigma-

Aldrich, Madrid, Spain). Membranes were resuspended in the corresponding buffer and were incubated (90 min at 30 °C) with the radioligand and each compound at a high concentration (40 µM) with the purpose of determining the % of radioligand displacement. Only in those cases in which radioligand displacement was greater than 70%, a complete competition curve with different compound concentrations (10−11−10−4 M) was carried out to obtain the Ki values. Nonspecific binding was determined with 10 µM WIN55212-2 (Sigma-Aldrich, Madrid, Spain) and total radioligand binding by incubation with the membranes in the absence of any compound. Filtration was performed by a Harvester filtermate (PerkinElmer, Boston, MA, USA) with Filtermat A GF/C filters pretreated with polyethylenimine 0.05%. After filtering, the filter was washed nine times with binding buffer and dried, and a melt-on scintillation sheet (MeltilexTM A, PerkinElmer, Boston, MA, USA) was melted onto it. Then, radioactivity was quantified by a liquid scintillation counter (Wallac MicroBeta Trilux, PerkinElmer, Boston, MA, USA). Competition binding data were analyzed by using GraphPad Prism, version 5.02 (GraphPad Software Inc., San Diego, CA, USA), and Ki values are expressed as the mean ± SEM of at least three experiments performed in triplicate for each.

2.2- *cAMP determination assays.* Determination of cAMP levels in HEK293 cells stably expressing the CB_2R was performed using the Lance-Ultra cAMP kit (PerkinElmer) according to the manufacturer's instructions. Briefly, HEK293 cells expressing the CB_2R were dispensed in white 384-well microplates at a density of 5.000 cells per well. Finally, cells were incubated for 60 min at room temperature with HTRF assay reagents, and fluorescence at 665 nm was analyzed on a PHERAstar Flagship microplate reader equipped with an HTRF optical module (BMG Labtech). Data analysis was made based on the fluorescence ratio emitted by the labeled cAMP probe (665 nm) over the light emitted by the europium cryptate-labeled anti-cAMP antibody (620 nm). A standard curve was used to calculate cAMP concentration. Forskolin stimulated cAMP levels were normalized to 100%. Data was analyzed by using

the GraphPad Prism program using nonlinear regression analysis. EC_{50} and E_{max} values are expressed as the mean ± SEM of at least three experiments performed in triplicate.

2.3- *CB_2R punctual mutations.* Mutations that alter the CB_2R sequence in one residue were produced using the QuikChange® Site-Directed Mutagenesis Kit and following the instructions of the manufacturar. Briefly, the cDNA for the human version of the cannabinoid CB2 receptor, cloned into pcDNA3.1, was amplified using sense and antisense primers harboring the triplets for the desired point mutation (Pfu turbo polymerase was used). The nonmutated DNA template was digested for 1 h with DpnI. PCR products were used to transform XL1-blue supercompetent cells. Finally, positive colonies were tested by sequencing to select those expressing the right DNA sequence.

3. Molecular modeling

3.1 *Docking studies.* Chromenopyrazole derivative A was docked into the orthosteric site using the structure of CB_2R in complex with Gi (PDB ID 6PT0),[2] and into the lipid-facing vestibule in TMs 1 and 7 using the Molecular Operating Environment (MOE) software (Chemical Computing Group Inc., Montreal, Quebec, Canada). The binding modes chromenopyrazole derivatives A and bitopic ligands **22, 25** and **27** were further studied by MD simulations.

3.2 *Molecular Dynamic Simulations.* Chromenopyrazole derivative A and bitopic ligands **22**, **25** and **27** bound to the CB_2R-G_i complex (PDB ID 6PT0) were embedded in a pre-equilibrated lipid bilayer box containing 1-palmitoyl-2-oleoyl-sn-glycero-3-phosphatidylcholine (POPC), water molecules (TIP3P) and monoatomic Na+ and Cl- ions (0.2 M). Assignment of ionization states and hydrogens at physiological pH for the selected structures was conducted with the Protonate3D method as implemented in MOE. Molecular systems were subject to a 1000 cycles of energy minimization, followed by 20 ns of gradual relaxation of positional restraints (corresponding to 100, 50, 25 and 10 $kJ.mol^{-1}nm^{-2}$) at protein backbone coordinates before the production phase in order to hydrate the receptor cavities and allow

lipids to pack around the protein. The AMBER99SB force field as implemented in GROMACS, Berger parameters for POPC lipids, and the general Amber force field (GAFF) with HF/6-31G*-derived RESP atomic charges for the ligands (see Figure S1) were used for the MD simulations. After equilibration, 500 ns (Figure S2) or two replicas of 1µs (Figure S5) of unrestrained MD simulation were performed at a constant temperature of 300 K using separate v-rescale thermostats for the receptor, ligands, lipids and solvent molecules. A time step of 2.0 fs was used for the integration of equations of motions. All bonds and angles were kept frozen using the LINCS algorithms. Lennard-Jones interactions were computed using a cutoff of 10 Å, and the electrostatic interactions were treated using PME with the same real-space cutoff under periodic boundary conditions. MD simulations were performed using GROMACS 2019.

References

1 J. Cumella, L. Hernández-Folgado, R. Girón, E. Sánchez, P. Morales, D. P. Hurst, M. Gómez-Cañas, M. Gómez-Ruiz, D. C. G. A. Pinto, P. Goya, P. H. Reggio, M. I. Martin, J. Fernández-Ruiz, A. M. S. Silva and N. Jagerovic, *ChemMedChem*, 2012, **7**, 452–463.

2 C. Xing, Y. Zhuang, C. Xing, Y. Zhuang, T. Xu, Z. Feng, X. E. Zhou, M. Chen and L. Wang, *Cell*, 2020, **180**, 645-654.e13.
